# *i-*GONAD (improved genome-editing via oviductal nucleic acids delivery), a convenient *in vivo* tool to produce genome-edited rats

**DOI:** 10.1038/s41598-018-30137-x

**Published:** 2018-08-13

**Authors:** Shuji Takabayashi, Takuya Aoshima, Katsuya Kabashima, Kazushi Aoto, Masato Ohtsuka, Masahiro Sato

**Affiliations:** 1 Laboratory Animal Facilities & Services, Preeminent Medical Photonics Education & Research Center, Hamamatsu University School of Medicine, 1-20-1 Handayama, Higashi-ku, Hamamatsu, Shizuoka 431-3192 Japan; 2 Department of Biochemistry, Hamamatsu University School of Medicine, 1-20-1 Handayama, Higashi-ku, Hamamatsu, Shizuoka 431-3192 Japan; 3 Department of Molecular Life Science, Division of Basic Molecular Science and Molecular Medicine, School of Medicine, Center for Matrix Biology and Medicine, Graduate School of Medicine, and The Institute of Medical Sciences, Tokai University, 143 Shimokasuya, Isehara, Kanagawa 259-1193 Japan; 4 Section of Gene Expression Regulation, Frontier Science Research Center, Kagoshima University, 8-35-1, Sakuragaoka, Kagoshima, Kagoshima, 890-8544 Japan

## Abstract

Zygote-microinjection or *in vitro* electroporation of isolated zygotes are now widely used methods to produce genome-edited mice. However, these technologies require laborious and time-consuming *ex vivo* handling of fertilized eggs, including zygote isolation, gene delivery into zygotes and embryo transfer into recipients. We recently developed an alternative method called improved genome-editing via oviductal nucleic acids delivery (*i*-GONAD), which does not require the above-mentioned *ex vivo* handing of zygotes, but instead involves intraoviductal instillation of genome-editing components, Cas9 protein and synthetic gRNAs, into the oviducts of pregnant females at the late 1-cell embryo stage under a dissecting microscope and subsequent electroporation. With this method, we succeeded in generating genome-edited mice at relatively high efficiencies (for example, knockout alleles were produced at ~97% efficiency). Here, we extended this improved technology to rats, and found that *i*-GONAD can create genome-edited rats in various strains, including Sprague Dawley and Lewis, and F1 hybrids (between Sprague Dawley and Brown Norway), with efficiencies of ~62% for indel mutations and ~9% for knock-ins. Thus, *i*-GONAD will be especially useful for the production of genome-edited rats in small laboratories where expensive micromanipulator systems and highly skilled personnel for embryo manipulation are unavailable.

## Introduction

Many bacterial clustered regularly interspaced short palindromic repeats (CRISPR)-associated (Cas) systems employ the dual RNA-guided DNA endonuclease, Cas9, to defend against invading phages and conjugative plasmids by introducing site-specific double-stranded breaks in target DNA^1,2^. This technology allows the precise manipulation of virtually any genomic sequence specified by a short stretch of guide RNA^3^. Recently, this CRISPR/Cas9-based genome editing system was simplified by development of a cloning-free CRISPR/Cas9, which allows for convenient genome editing by eliminating the time-consuming engineering of individual guide RNA expression vectors^4^. This system uses a synthetic single-guide RNA (sgRNA) that mimics the natural dual *trans*-activating CRISPR RNA (tracrRNA)-CRISPR RNA (crRNA) structure. This system is now commercially available through custom RNA synthesis services at many companies^4^. To create genome-edited animals, direct injection of CRISPR/Cas9-related nucleic acids into pronuclear stage embryos, termed “zygote microinjection”, has been widely used^5–8^. This approach requires several time-consuming and laborious steps, similar to microinjection-based transgenesis, such as *ex vivo* handling of embryos (isolation of zygotes, microinjection of nucleic acids into embryos, brief cultivation of the injected embryos and egg transfer to recipient females). The nucleic acid delivery method of microinjection can be bypassed by using *in vitro* electroporation of the components with the isolated eggs^9–13^. However, this electroporation-based technology still requires isolation of zygotes, brief cultivation of the electroporated eggs and subsequent transfer to recipient females to allow them to develop further.

Genome-editing via oviductal nucleic acid delivery (GONAD) was first developed as a method of *in situ* genome editing of preimplantation embryos present within an oviduct lumen^14,15^. This method employs intraoviductal instillation of genome editing components and subsequent *in vivo* electroporation of the oviduct and, therefore, does not require any of *ex vivo* handling of preimplantation embryos, which is required for the production of genome-edited mice using zygote microinjection and *in vitro* electroporation. With GONAD, simple insertion/deletion (indels) mutations were achieved in the resulting offspring at a rate of 28~31%, when 2-cell embryos [at 1.5 days postcoitum (dpc)] were targeted and mRNA for Cas9 and gRNA was introduced. Unfortunately, a relatively high rate of mosaicism was experienced in these offspring^14^. To circumvent this problem, we tested if 0.7 dpc, which corresponds to the zygote stage, is suitable for GONAD because most cumulus cells that potentially hamper the uptake of intraoviductal-instilled nucleic acids are thought to be detached at this stage. Furthermore, we tested the possible usefulness of ribonucleoprotein (RNP) complexes, which consist of Cas9 nuclease complexed with synthetic sgRNA (or annealed crRNA and tracrRNA). As a result, we succeeded in obtaining mice with indels in their genome at relatively high efficiencies (~97%), as well as mice with large deletions in a target gene, and with 50% knock-in (KI) of a desired sequence into a target locus^16,17^. We re-named the improved version of the method as “improved GONAD (*i-*GONAD)”^16^. Until recently, the *i*-GONAD method has been demonstrated in mouse strains such as ICR, C3H/HeN, C57BL6/N, DBA2 and hybrids (B6C3F1 and B6D2F1).

In this study, we tested if *i-*GONAD can be applied to other small animal models, such as rats. For this purpose, we tested *i*-GONAD in three rat strains: albino Sprague Dawley (SD), albino Lewis (LEW), and pigmented Brown Norway (BN). We examined whether (1) CRISPR/Cas9-mediated induction of indels is possible at the wild-type *Tyr* locus, (2) whether CRISPR/Cas9-mediated KI of a single stranded (ss) oligonucleotide (ODN) into the *Tyr* mutation is possible in SD and LEW rats because the albino mutation has been identified as the Arg299His missense mutation in exon 2 of the tyrosinase (*Tyr*) gene^18^, and (3) whether the acquired phenotype is transmitted to the next generation. Furthermore, we attempted to use *i*-GONAD to disrupt the endogenous *Pax6* locus, which is involved in the formation of eyes and nasal structures during embryogenesis^19,20^.

## Results

### Preliminary test for successful delivery of exogenous substances to preimplantation rat embryos using *i*-GONAD

**Figure 1:**
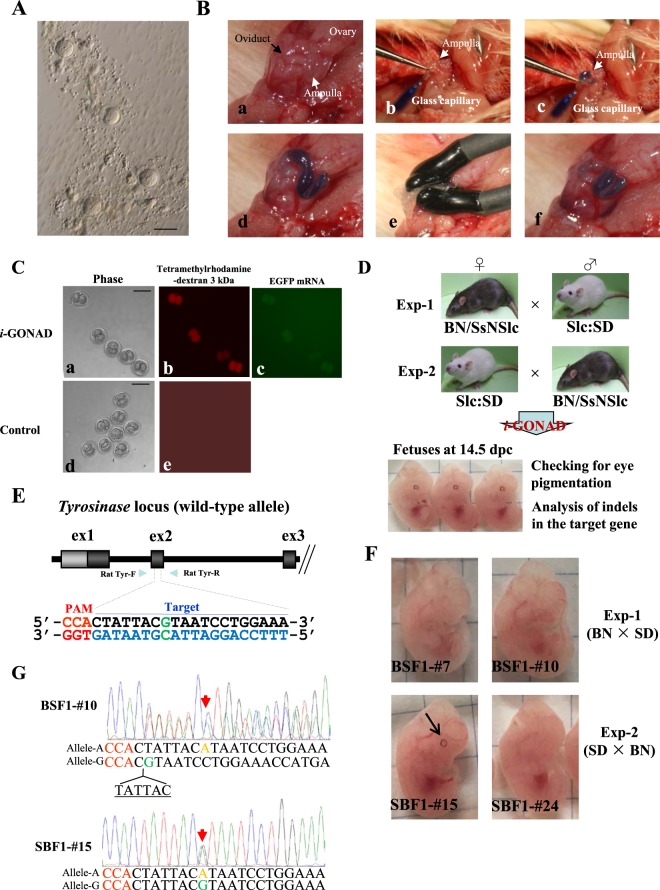
(**A**) One-cell rat embryos isolated from 0.7 dpc female SD rats. Note the presence of cumulus cell-free 1-cell embryos. Bar = 100 μm. (**B**) The *i*-GONAD procedure. Under appropriate anesthesia, ovary/oviduct/uterus of a pregnant rat at 0.7 dpc was exposed under a dissecting microscope. After positioning the oviduct, it was gently grasped in forceps (a). A micropipette was then inserted into the lumen of the oviduct by piercing the oviduct wall and (controlled by a mouthpiece) approximately ~1.5 µL solution was immediately expelled into the oviduct (b). The injected substances can be easily identified by the co-injected Fast Green (c,d). After instillation, the entire oviduct was covered with a piece of wet Kimwipe towel and then subjected to *in vivo* electroporation using tweezer-type electrodes (e). After removal of electrodes, the electroporated area remains intact (f). (**C**) Detection of fluorescent 2-cell embryos after *i*-GONAD. When pregnant (0.7 dpc) SD rats were subjected to *in vivo* electroporation after intraoviductal instillation of Rhodamine + EGFP mRNA-containing solution (*i*-GONAD), some of the recovered 2-cell embryos exhibited distinct red and/or green fluorescence (a–c). In contrast, intraoviductal instillation of Rhodamine alone (without electroporation) (Control) resulted in embryos showing no fluorescence (d,e). Bar = 100 μm. (**D**) Flowchart of experiments to test the feasibility of *i-*GONAD for the production of mid-gestational fetuses carrying indels at a single target locus (*Tyr*). In Exp-1, BN females were mated to SD males to obtain pregnant BN rats, while in Exp-2, SD females were mated to BN males to obtain pregnant SD rats. (**E**) Schematic illustration of the wild-type *Tyr* locus. The target sequence of *Tyr* exon 2 (ex2) recognized by gRNA is overlined and the PAM sequence is marked in red. The target nucleotide “G” marked in green is a key nucleotide for tyrosinase activity; nucleotide replacement at this position often causes an albino phenotype. Primers for amplification 598 bp of exon 2, Rat Tyr-F and -R, are shown. (**F**) Offspring obtained after *i-*GONAD in Exp-1 or -2. The embryos numbered BSF1-#7, BSF1-#10 and SBF1-#24 exhibited non-pigmented eyes. The fetus numbered SBF1-#15 had pigmented eyes (shown by an arrow), probably as a result of unsuccessful genome editing at the *Tyr* locus. (**G**) Direct sequencing of PCR products derived from the embryos shown in (**F**). Red arrow in BSF1-#10, which showed eye non-pigmentation, indicate heterozygous bi-allelic KO at the *Tyr* target (nucleotide “G” marked in green in **E**) because one allele (called allele-A) has the nucleotide “A” (shown in brown) and the other allele (called wild-type allele or allele-G) lacks 6-bp sequence above the PAM. In contrast, the red arrow in SBF1-#15 indicates heterozygous mono-allelic KO, because one allele (called allele-A) has the nucleotide “A” (shown in brown), whereas the wild-type nucleotide “G” exists at the same position as allele-G.

Before testing the possibility of successful *i*-GONAD in rat embryos, we first examined whether late 1-cell eggs isolated at 0.7 dpc are free from cumulus cells, which act as a barrier to the delivery of exogenous substances into embryos^16^. As shown in Fig. 1A, the recovered 0.7 dpc rat SD embryos were indeed free from cumulus cells. We next examined whether rat 0.7 dpc embryos can take up a substance instilled into an oviduct lumen after *in vivo* electroporation. For this, we instilled about 1.5 μL of a solution containing enhanced green fluorescent protein (EGFP) mRNA, tetramethylrhodamine-dextran (Rhodamine) and Fast Green into the oviduct lumen of SD females at 0.7 dpc (Fig. 1B-a–d), and subsequently performed *in vivo* electroporation using a NEPA21 electroporator (Fig. 1B-e). Rhodamine is used for evaluating gene delivery in rat embryos upon *in vitro* electroporation^9^. The electroporated portion of the oviduct appeared to be intact immediately after electroporation (Fig. 1B-f). One day after *i*-GONAD, 2-cell eggs were isolated from these treated females and inspected for fluorescence. Of the 20 *i*-GONAD-treated embryos recovered, 17 exhibited red fluorescence and nine exhibited green fluorescence (Fig. 1C-a~c and Table 1). In contrast, no fluorescent embryos were detected when intraoviductal instillation of the solution alone (without electroporation) was performed (Fig. 1C-d,e and Table 1). These findings indicate that *i*-GONAD is feasible in 0.7 dpc rats.

**Table 1:** Summary of the *i*-GONAD procedure shown in Fig. 1C.

Treatment	No. rats treated	No. pregnant rats	No. 2-cell embryos collected	No. 2-cell embryos showing red fluorescence (%)	No. 2-cell embryos showing green fluorescence (%)
*i*-GONAD	3	2	20	17 (85)	9 (45)
Control (without electroporation)	2	2	17	0 (0)	ND

ND: Not done.

### Induction of indels in the *Tyr* locus in pigmented rats

To test whether *i*-GONAD is useful for the production of indel-based KO rats, we decided to mutate the *Tyr* locus (exon 2), because this causes non-pigmentation (albinism) of the eyes and coat in mice and rats^18,21^. For this purpose, F1 hybrids obtained from mating between BN and SD rats were used for *i*-GONAD because they exhibit pigmentation in their eyes and coat due to expression of normal tyrosinase from a wild-type *Tyr* allele. They can be produced by mating between pigmented BN females and albino SD males or between SD females and BN males (Fig. 1D). Sequencing of *Tyr* from these F1 hybrids revealed a heterozygous genotype, of a wild-type allele (hereafter called “allele-G”) and a mutant allele (hereafter called “allele-A”) (data not shown). Intraoviductal instillation of Cas9 protein complexed with crRNA:tracrRNAs targeted to wild-type *Tyr* (Fig. 1E) was carried out on late 1-cell F1 hybrid eggs at 0.7 dpc and *in vivo* electroporation performed. When *i*-GONAD was carried out on five BN females that had been mated to SD males [Experiment-1 (Exp-1); Fig. 1D], three became pregnant, and gave rise 13 mid-gestational fetuses (Table 2). Of these 13 fetuses, eight (62%) had non-pigmented eyes (as exemplified by BSF1-#7 and BSF1-#10 in Fig. 1F and Table 2). Sequencing of PCR products spanning *Tyr* exon 2 demonstrated that all fetuses showing non-pigmentation had indels mutations (as exemplified by the BSF1-#7 fetus shown in Supplementary Table S1). Furthermore, the BSF1-#10 fetus exhibited a 6 bp deletion (TATTAC) just upstream of the protospacer adjacent motif (PAM) sequence in the wild-type allele-G (Fig. 1G and Supplementary Table S1). Other indels included deletion of a sequence (four fetuses, BSF1-#4, 5, 10 and 13), insertion of nucleotide(s) (two fetuses, BSF1-#7 and 9), insertion and deletion of nucleotides (one fetus, BSF1-#6) and replacement of nucleotides (one fetus, BSF1-#3) (Tables 2 and S1). Notably, no indels were noted in fetuses showing pigmented eyes (Supplementary Table S1).

**Table 2:** Summary of the fetal offspring obtained through Exp-1 and -2.

Experiment	No. pregnant rats/no. rats treated	No. fetuses obtained	No. fetuses carrying de-pigmented eyes (%)	Fetuses with modified alleles		
				No. homozygous or compound heterozygous bi-allelic mutation (%)	No. heterozygous bi-allelic mutation (%)	No. mosaic mutation (%)
Exp-1 (mating between female BN and male SD)	3/5	13	8 (62)	2 (25)	6 (75)	0 (0)
Exp-2 (mating between female SD and male BN)	3/3	16	9 (56)	1 (11)	5 (56)	3 (33)

In Experiment-2 (Exp-2, Fig. 1D), three *i*-GONAD-treated SD females that had been mated with BN males produced a total of 16 fetuses (Table 2). Of these, nine (56%) exhibited non-pigmented eyes (as exemplified by fetus SBF1-#24 in Fig. 1F and Table 2). All of the fetuses with non-pigmented eyes had indels in the wild-type allele-G (between the target G nucleotide and the PAM). For example, fetus SBF1-#24 had insertion of two nucleotides (AA) between the target nucleotide G and the PAM (Supplementary Table S2). In contrast, fetuses showing pigmented eyes (indicated by an arrow in fetus SBF1-#15 in Fig. 1F) had both wild-type allele-G and mutated allele-A in the *Tyr* locus (indicated by red arrows in Fig. 1G and Supplementary Table S2). Similar to Exp-1, mutations found in all of the resulting fetuses were classified as deletion of a sequence (three fetuses, SBF1-#16, 22 and 23), insertion of nucleotide(s) (two fetuses, SBF1-#24 and 26), insertion and deletion of nucleotides (one fetus, SBF1-#25) and mosaic mutation (three fetuses, SBF1-#27, 28 and 29), which are shown by at least three types of ideogram (Tables 2 and S2). Notably, two of nine fetuses carrying indels had mutations in the albino-specific allele: for example, one was mosaic and the other had mutations in both alleles (Supplementary Table S2).

### Single nucleotide edition at the *Tyr* locus in albino LEW and SD rats

**Figure 2:**
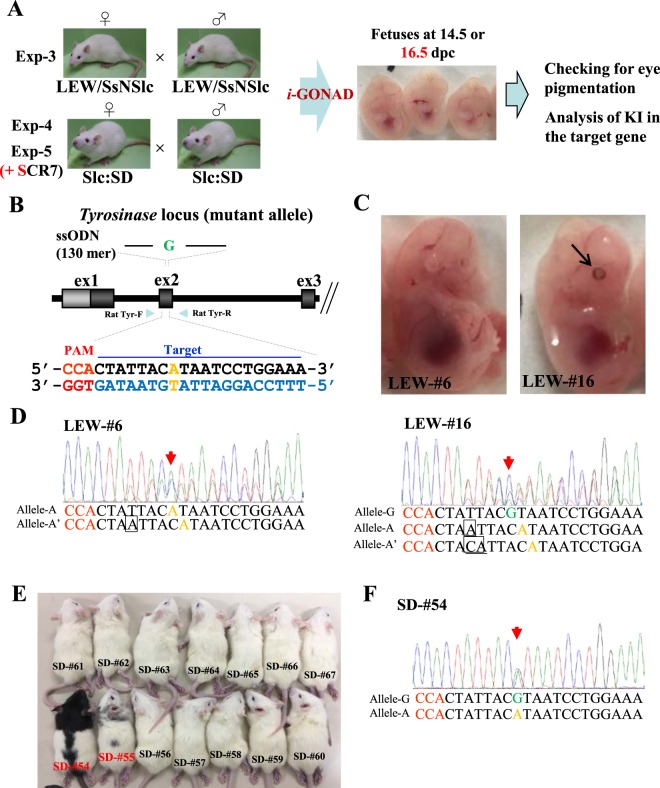
(**A**) Flowchart of the experiments used to test the feasibility of *i-*GONAD for the production of rats with ssODN-mediated KI at a single target locus (*Tyr*). In Exp-3, LEW females were mated to LEW males to obtain pregnant LEW rats. LEW is an albino strain with non-pigmented eyes. In Exp-4 and 5, SD females were mated to SD males to obtain pregnant SD rats. SD is an albino strain with non-pigmented eyes and coat. NHEJ-inhibitor, SCR7, was co-injected with RNP + ssODN in Exp-5. (**B**) Schematic illustration of the mutated *Tyr* locus. The target sequence of *Tyr* exon 2 (ex2) recognized by gRNA is overlined and the PAM sequence is marked in red. The nucleotide “A” marked in brown is a mutated nucleotide that causes the albino phenotype. Primers for amplification 598 bp of exon 2, Rat Tyr-F and -R, are shown. In the ssODN, the wild-type nucleotide “G” that corresponds to the mutated nucleotide “A” is shown in green. (**C**) Offspring obtained after *i-*GONAD of pregnant LEW females. The fetuses numbered LEW-#6 had albino eyes, while the fetus numbered LEW-#16 had pigmented eyes (shown by an arrow), probably as a result of successful KI of the ssODN into the *Tyr* locus. (**D**) Direct sequencing of PCR products derived from the embryos shown in (**C**). Red arrows in LEW-#6, which has non-pigmented eyes, indicate heterozygous bi-allelic mutations at the *Tyr* target, because one allele (called allele-A) has the nucleotide “A” (shown in brown) and the other allele (called allele-A’) has one nucleotide insertion above the PAM in allele-A. In contrast, the red arrow in LEW-#16 indicates a mosaic pattern of electrophoretograms, in which at least three nucleotides are present at the target site, including the wild-type G (shown in green) in one allele (called allele-G). Nucleotide(s) enclosed by boxes are those inserted. (**E**) Offspring obtained after *i-*GONAD of pregnant SD females. The two offspring numbered SD-#54 and -#55 showed pigmented coat color, but the other rats had albino coats. (**F**) Direct sequencing of PCR products derived from the animals shown in (**E**). Red arrows in SD-#54, which has pigmented eyes and/or coat, indicate the presence of a nucleotide “G” (shown in green) at the *Tyr* target in one allele (called allele-G).

In Experiment-3 (Exp-3), we examined whether the albino phenotype in LEW rats can be rescued through *i*-GONAD-based KI using an ssODN and Cas9 protein complexed with crRNA:tracrRNAs targeted to the mutated *Tyr* gene (Fig. 2A,B). Of seven LEW females treated with *i*-GONAD, five became pregnant, and generated a total of 22 mid-gestational fetuses (Table 3). One fetus (termed LEW-#16) (5%) exhibited pigmentation in its eye (arrow in Fig. 2C). Sequence analysis of PCR products from genomic DNA and from sub-cloned plasmid DNA demonstrated that the mutations found in the *Tyr* locus of the LEW-#16 fetus were mosaic. One allele (termed allele-G) had a corrected G nucleotide that was created after ssODN-based KI, while the other albino-specific alleles (termed allele-A and A’) had insertion of nucleotides such as A or CA upstream of the PAM (Fig. 2D and Supplementary Table S3). Sequencing of the junction between endogenous *Tyr* and the ssODN revealed that the ssODN was inserted correctly into the target site (Supplementary Fig. S1). Nine (41%) of 22 fetuses tested had mutations in the albino-specific allele (as exemplified by LEW-#6 in Fig. 2D; Tables 3 and S3). Of nine samples showing indels, four had mutations in one allele (heterozygous mutation, LEW-#4, -#6, -#14 and -#15), three in both alleles (Compound heterozygous mutations, LEW-#3, -#12 and -#21) and two showed mosaic mutations (LEW-#16 and -#20), of which one exhibited KI, as mentioned above (Supplementary Table S3). No fetuses showing albino pigmentation had a KI genotype.

**Table 3:** Summary of the offspring obtained through *i*-GONAD-based KI.

Experiment	Treatment	No. pregnant rats/no. rats treated	No. fetuses/pups obtained	Fetuses/pups with modified alleles		
				No. indels (%)	No. KI allele (%)	No. mosaic alleles (%)
Exp-3 (*i*-GONAD using LEW strain)	Cas9/tracr/crRNA + ssODN	5/7	22	9 (41)	1 (5)	2 (9)
Exp-4 (*i*-GONAD using SD strain)	Cas9/tracr/crRNA + ssODN	4/7	40	16 (40)	2 (5)	5 (13)
Exp-5 (*i*-GONAD using SD strain)	Cas9/tracr/crRNA + ssODN + 1 μM SCR7	6/6	27	11 (41)	2 (7)	6 (22)
Exp-5 (*i*-GONAD using SD strain)	Cas9/tracr/crRNA + ssODN + 10 μM SCR7	3/5	21	10 (48)	2 (9)	5 (24)

Similar to Exp-3, in Experiment-4 (Exp-4) we examined whether the albino phenotype in SD rats can be rescued by *i*-GONAD-based genome editing (Fig. 2A). After *i-*GONAD, mid-gestational fetuses were dissected and checked for eye pigmentation. In some cases, *i*-GONAD-treated females were allowed to deliver their pups. Of seven SD females treated, four were pregnant, and generated a total of 23 fetuses and 17 newborn rats (Table 3). None of the resulting fetuses showed pigmented eyes (data not shown), while two newborns (SD-#54 and -#55) had pigmented eyes together with pigmented coat (Fig. 2E). These two pigmented individuals were healthy throughout their life. Direct sequencing of PCR products derived from SD-#54 demonstrated that in one allele (termed allele-G) insertion of the target nucleotide “G” derived from the ssODN was detectable, while in the other allele (termed allele-A) the presence of indels was discernible (Fig. 2F and Supplementary Table S4). Taken together, the total number of individuals showing successful KI was two, providing a KI efficiency of 5% (Table 3). Of 40 individuals obtained, 16 had indels [eight for one allele (SD-#5, -#8, -#9, -#19, -#23, -#55, -#56 and -#67), three for both alleles (SD-#11, -#18 and -#26) and five for mosaic mutations(SD-#7, -#15, -#16, -#20 and -#58)] in the target sequence (Tables 3 and S4).

As mentioned above, *i*-GONAD-mediated KI efficiency in rats was as low as ~5%. To improve this low KI efficiency, we added 1 or 10 μM SCR7 in Experiment-5 (Exp-5) (Fig. 2A), a selective and potent inhibitor of non-homologous end joining (NHEJ)^22,23^, into the RNP-containing solution prior to *i*-GONAD. It is expected that addition of SCR7 will increase the rate of KI *via* HR (homologous recombination) when an ssODN is used as an HR donor template. Of six SD female rats subjected to *i*-GONAD in the presence of 1 μM SCR7, six became pregnant, and generated a total of 27 mid-gestational fetuses (Table 3). Of these, two (7%) (termed SD-#27 and -#46) had pigmented eyes (Tables 3 and S5). *i*-GONAD in the presence of 10 μM SCR7 yielded 21 fetuses, of which two (9%) (termed SD-#75 and -#76) exhibited pigmentation in the eyes (Tables 3 and S6). Sequencing of the PCR products derived from these pigmented fetuses (SD-#27, -#46, -#75 and -#76) demonstrated that all of the samples have a target nucleotide “G” that is considered to be derived from the ssODN (Supplementary Tables S5 and S6). The other albino fetuses did not show KI-mediated insertion of ssODN. In total, 11 of 27 fetuses (41%) using 1 μM SCR7 and 10 of 21 fetuses (48%) using 10 μM SCR7 were successfully genome-edited, and only 7 to 9% exhibited successful KI (Tables 3, S5 and S6), indicating that addition of SCR7 (even at 10 μM) into the RNP-containing solution appeared not to affect *i*-GONAD-mediated KI efficiency in rats.

### Successful transmission of modified traits into the next generation

**Figure 3:**
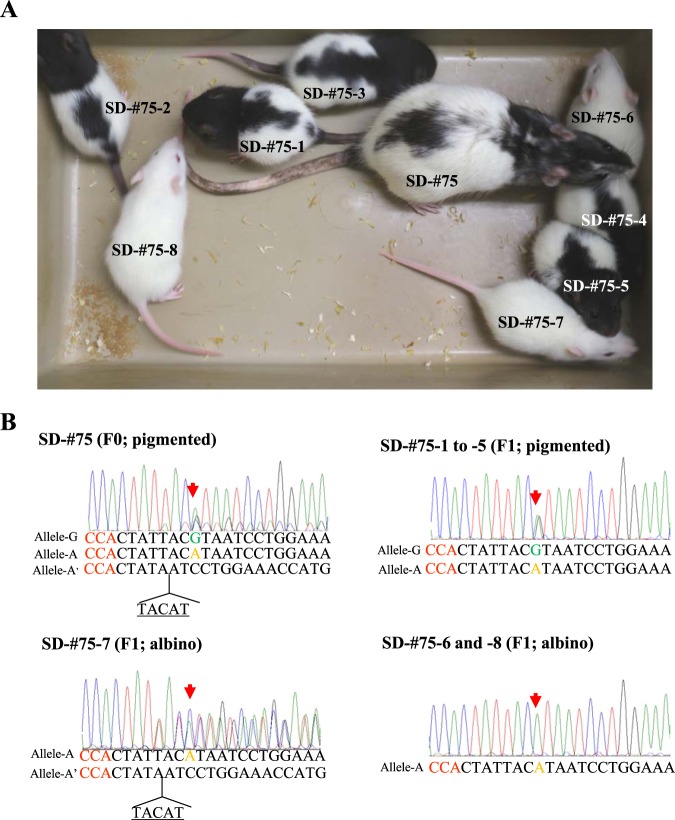
(**A**) F1 offspring (SD-#75-1 to -8) of SD-#75 F0 female (showing pigmentation) after mating with unedited F0 male SD littermates. (**B**) Direct sequencing of PCR products derived from the animals is shown in (**A**). Red arrows indicate the position that is important for tyrosinase function. Note that the modified traits in the *Tyr* locus found in the F0 mouse (SD-#75) are successfully transmitted to the F1 offspring (including SD-#75-1 to -5 and -7).

To test for germ-line transmission of mutated alleles obtained in rats through *i*-GONAD, we obtained several F1 offspring by cross-breeding the SD-#75 F0 male (showing a pigmented phenotype after successful ssODN KI into the target *Tyr* locus; Supplementary Table S6) to unedited albino SD F0 rats (females). Of eight pups obtained five (termed SD-#75-1 to 5) exhibited pigmentation in both eyes and coat (Fig. 3A). Notably, the pigmented pattern of these F1 offspring was similar to that of its parent, SD-#75 (Fig. 3A). Sequence analysis of genomic DNA isolated from SD-#75 revealed that one allele (termed allele-G) had a corrected G nucleotide that was created after ssODN-based KI, while the other albino-specific alleles (termed allele-A and A’) were normal (allele-A) or had deletion of five nucleotides upstream of the PAM (allele-A’) (Fig. 3B). All of the pigmented F1 offspring, SD-#75-1 to -5, had allele-G and -A (Fig. 3B). One albino F1 offspring SD-#75-7 had allele-A and -A’ (Fig. 3B), while the other two albino F1 offspring, SD-#75-6 and -8, had allele-A alone (Fig. 3B). Thus, all types of modified alleles (allele-G and -A’) found in the F0 parent were successfully transmitted to the F1 generation. These results clearly indicate that it is possible to create genome-edited rat lines using *i*-GONAD.

### Induction of indels in the *Pax6* locus of hybrids between female SD and male BN rats

**Figure 4:**
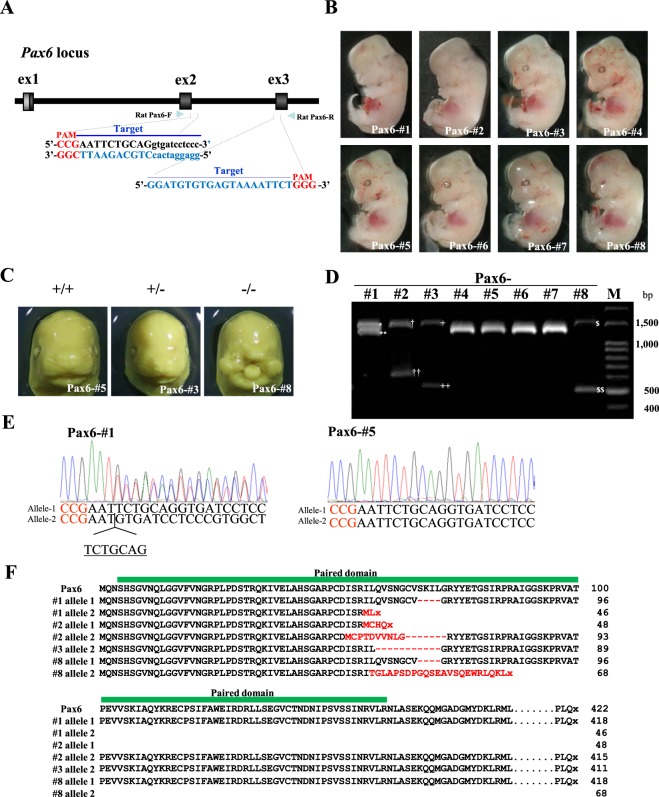
(**A**) Schematic illustration of the wild-type *Pax6* locus. Exons (ex1 to ex3) and introns are indicated by black boxes and a black line, respectively. Dual guide RNAs were used on the *Pax6* locus: namely, the two target sequences recognized by gRNA are overlined and the PAM sequences are shown in red. The position of primers (Rat Pax6-F and -R) used to identify indels around the target sequences is indicated by arrowheads. (**B**) Fetal offspring (Pax6-#1 to #8) at 16.5 dpc obtained after *i*-GONAD targeted towards the paired domain of *Pax6*. Various abnormal phenotypes such as deformity of the facial structure together with loss of eye cup (as exemplified by Pax6-#1, #2 and #8) are discernible. (**C**) Representative 16.5 dpc fetuses exhibiting various types of craniofacial structure. Fetus Pax6-#5 and -#3 exhibited normal facial structure, and was wild-type and heterozygous for *Pax6*, respectively. Fetus Pax6-#8 completely lacked eyes and the lateral nasal prominence, and was homozygous knockout for *Pax6*. (**D**) Genotyping of the fetuses shown in (**B**). Fetuses Pax6-#1 to #3 and #8 have heterozygous PCR products (*allele-1of Pax6-#1, **allele-2 of Pax6-#1, ^†^allele-1of Pax6-#2, ^††^allele-2 of Pax6-#2, ^+^allele-1of Pax6-#3, ^++^allele-2 of Pax6-#3, ^$^allele-1of Pax6-#8 and ^$$^allele-2 of Pax6-#8). Fetuses Pax6-#4 to #7 have wild-type PCR products (1,489 bp). (**E**) Direct sequencing of PCR products derived from the animals shown in (**B**). Fetus Pax6-#5, which was judged as wild-type through morphological inspection (shown in **B** and **C**) and genotyping (shown in **D**), exhibited normal sequence for *Pax6*. In contrast, fetus Pax6-#1, which was judged as KO through morphological inspection (shown in **B**) and genotyping (shown in **D**), exhibited a 7 bp sequence deletion (underlined) in front of the PAM (shown in red). (**F**) Comparison of PAX6 amino acid sequences from fetuses derived from SD females subjected to *i*-GONAD-mediated induction of indels at the *Pax6* locus. The control amino acid sequence of wild-type PAX6 protein is shown on the top. The amino acid sequences of the mutated PAX6 proteins (derived from Pax6-#1 to #3 and #8) are shown. Predicted consequences of the mutation on amino acid sequences are highlighted in red. Stop codons are shown as x. The green bar indicates the paired domain.

To test whether *i*-GONAD is useful for genome editing towards other loci besides *Tyr*, we next decided to knock out the paired domain of *Pax6* in rats in Experiment-6 (Exp-6). Ablation of *Pax6* is known to causes developmental abnormalities in eyes and nasal structures in mice and rats^20,24^. For this experiment, we used pregnant albino SD females mated to pigmented BN males, because all resultant fetuses should have pigmented eyes (Fig. 1D). Intraoviductal instillation of Cas9 protein complexed with the two crRNA:tracrRNA complexes targeted to the wild-type *Pax6* (Fig. 4A) was carried out on late 1-cell embryos (F1 hybrid embryos between SD and BN) at 0.7 dpc prior to *in vivo* electroporation. Of three SD females, two became pregnant and gave rise to a total of eight mid-gestational fetuses (Fig. 4B and Table 4). Three of these fetuses (termed #1, #2 and #8) (38%) had eye and nasal defects (Fig. 4B and Table 4). These abnormal fetuses completely lacked eyes and the lateral nasal prominence (#8 in Fig. 4C).

**Table 4:** Summary of the fetuses obtained through *i*-GONAD-based induction of indels in the target locus *Pax6* (Exp-6).

Experiment	No. pregnant rats/no. rats treated	No. fetuses obtained	No. fetuses carrying abnormal eyes and nose (%)	Fetuses with modified alleles		
				No. compound heterozygous bi-allelic mutation (%)	No. heterozygous bi-allelic mutation (%)	No. mosaic mutation (%)
Exp-6 (mating between female SD and male BN)	2/3	8	3 (38)	3 (38)	1 (13)	0 (0)

PCR amplification spanning exons 2 and 3 of *Pax6* revealed unexpected heterozygous bands in fetuses Pax6-#1, -#2, -#3 and -#8, while fetuses Pax6-#4 to -#7 had the expected single PCR band of 1,489 bp (Fig. 4D). PCR using unedited samples (including SD, BN, and SBF1 offspring) also demonstrated the presence of a single band of 1,489 bp (Supplementary Fig. S2). To examine the unexpected heterozygous bands in fetuses Pax6-#1, -#2, -#3 and -#8 in more detail, the PCR products were sub-cloned into a pTA cloning vector. Sequencing of the PCR product cloned into a pTA cloning vector demonstrated that all fetuses showing an abnormal phenotype had indels in the target *Pax6* locus (Figs 4E and S3). For example, fetus Pax6-#1 had a 12 bp deletion in one allele (called “allele-1 of Pax6-#1” in Supplementary Fig. S3; shown by * in Pax6-#1 of Fig. 4D) and a 7 bp (TCTGCAG) deletion and a 158 bp deletion in another allele (called allele-2 of Pax6-#1 in Figs 4E and S3 shown by ** in Pax6-#1 of Fig. 4D). Fetus Pax6-#2 exhibited a 9 bp (GTCAGGTGA) insertion and a 231 bp deletion in one allele (called “allele 1 of Pax6-#2”; shown by † in Pax6-#2 of Figs 4D and S3) and also a 860 bp deletion and a 2 bp (AT) deletion in another allele (called “allele 2 of Pax6-#2”; shown by †† in #2 of Figs 4D and S3). Fetus Pax6-#3 showed normal morphology (Fig. 4B,C) and had a 947 bp deletion in one allele (called “allele 2 of Pax6-#3”; shown by ++ in Pax6-#3 of Figs 4D and S3), but had normal sequence in the other allele (called “allele 1 of Pax6-#3”; shown by + in Pax6-#3 of Figs 4D and S3). Fetus Pax6-#8 had a 12 bp deletion in one allele (called “allele 1 of Pax6-#8”; shown by $ in Pax6-#8 of Figs 4D and S3), and a 960 bp deletion and a 1 bp replacement) in the other allele (called “allele 2 of Pax6-#8”; shown by $$ in Pax6-#8 of Figs 4D and S3). Notably, one allele (allele 1of Pax6-#1) in fetus Pax6-#1 was the same as that found in fetus Pax6-#8 (allele 1 of Pax6-#8). As shown in Supplementary Fig. S3 the DNA sequences of allele 2 of Pax6-#1, allele 1 of Pax6-#2 and allele 2 of Pax6-#8 had a premature stop codon, which will result in premature termination of protein synthesis leading to formation of an abnormal PAX6 protein (Fig. 4F). Furthermore, sequence analysis of allele 1 of Pax6-#1, allele 2 of Pax6-#2, allele 2 of Pax6-#3, and allele 1 of Pax6-#8 indicates coding of a PAX6 protein that lacks part of the PAX6 paired domain (Fig. 4F).

### Off-target analysis

Off-target cleavage in genome-edited animals is considered as one of the most serious problems associated with CRISPR/Cas9-mediated genome editing^25–28^; therefore we assessed this problem by checking off-target candidate loci containing up to 3-bp mismatches compared with the 20-bp guide sequence of each crRNA, as shown in Supplementary Table S7 [W-off target 1 (*Fryl*) for BSF1-#3, 6, 7, 7, 10 and 13, and SBF1-#13, 22, 23, 24, 27 and 28; M-off target 1, 2, 3 (*Fryl*) and 4 for LEW-#3, 4, 6, 12, 16, 21 and SD-#5, 15, 27, 46, 54 and 55; P1-off target 1 (*Cyp4a1*) and P2-off target 1, 2, 3 and 4 (*Dis3I2*) for Pax6-#1, 2, 3 and 8]. We failed to detect any sign of off-target digestion at any of the off-target candidate loci in KI or KO rats, (Supplementary Fig. S4).

## Discussion

Genome editing in rats has been successful using zygote microinjection of genome editing components or *in vitro* electroporation of zygotes with these components^9,29–31^. In this work we successfully demonstrated that genome-edited rats can be achieved by *i*-GONAD. Recently, Kobayashi *et al*.^32^ showed successful GONAD in Wistar Kyoto (WKY) rats using the NEPA21 electroporator system. When compared with *i*-GONAD/GONAD performed in mice^14,16^, the procedure presented here is almost the same with respect to the amount of RNP-containing solution injected intraoviductally, and the *in vivo* electroporation conditions used, which were Poring pulse: 50 V/5 msec pulse/50 msec pulse interval/3 pulses/10% decay (±pulse orientation) and Transfer pulse: 10 V/50 msec pulse/50 msec pulse interval/3 pulses/40% decay (±pulse orientation). Kobayashi *et al*.^32^ explored optimal conditions for GONAD by changing electroporation parameters and finally employed the following conditions, Poring pulse: 50 V/5 msec pulse/50 msec pulse interval/3 pulses/10% decay (±pulse orientation) and Transfer pulse: 10 V/50 msec pulse/50 msec pulse interval/6 pulses/40% decay (±pulse orientation). Notably, Takahashi *et al*.^14^ and Kobayashi *et al*.^32^ employed trypan blue (used to monitor successful oviductal instillation of a solution), but we found that Fast Green is a preferable alternative to trypan blue, because it did not form any visible aggregates (unlike trypan blue) when mixed with RNP (data not shown).

In this study, we achieved relatively high efficiencies (~62%) for inducing indels in the target gene when RNP-based GONAD was applied to various rats. These rates appear to be comparable to those reported for mice (~97%)^16,17^ and rats (~59%)^32^. Furthermore, the rate of occurrence of mosaic mutations appears to be low in our system. Total mosaic rate was 33% (21 mosaic mutations/63 indels mutations, Tables 2 and 3). The direct sequencing of PCR products produced electrophoretograms showing patterns for homozygous bi-allelic or heterozygous mono-allelic KO (Fig. 1G), and almost no electrophoretograms showed overlapping or erroneous electrophoretograms as shown by NNN.

The conditions employed here were effective for genome editing in rats from various strains. For example, the rates of indels induced by *i*-GONAD using SD (closed colony) and LEW (inbred) rats were 40 and 41%, respectively. Furthermore, we found that one of eight fetuses (13%) from F344 (inbred) rats or six of 14 fetuses (43%) from WKY (inbred) rats had indels for genes X and Y, respectively (unpublished data). Unfortunately, we failed to obtain genome-edited offspring (fetuses/newborns) from the BN females that had been successfully mated to BN males, despite repeated *i*-GONAD trials (seven times in total). The reason why *i-*GONAD fails when BN strains are used remains unknown. Similarly, in mice there is a strain-difference regarding the efficiency of mutation induction by *i-*GONAD. For example, ICR females (closed colony) delivered viable fetuses with indels at an efficiency of 84%, while C57BL/6 inbred females failed to deliver pups for *Tyr* gene editing (Supplementary data from Ohtsuka *et al*.)^16^. In this context, it may be strictly required to determine the optimal conditions that allow survival of embryos/fetuses with successful *i*-GONAD-mediated genome editing for each rodent strain.

The efficiency of *i*-GONAD-based KI using RNP + ssODN was lower than that for indel-based mutation induction (about 5% *vs*. 40%; Table 3). This appears to be consistent with the situation in mice (50% for KI *vs*. 97% for indels)^16^. Notably, it has been suggested that low KI efficiency in cultured cells after transfection with genome editing components can be overcome by the combined use of genome-editing components and NHEJ inhibitors, such a SCR7^22,23^. Unfortunately, addition of SCR7 (1 or 10 μM) to intraoviductal injections failed to greatly improve the KI efficiency (see Table 3), despite its concentration being increased by up to 4~5-fold in comparison to the levels used for *in vitro* transfection^23^. Addition of highler concentrations of SCR7 (for example, 100 μM) may be required to improve the *i*-GONAD-based KI system. New reagents, including RS-1 and L755, 507^33,34^, have shown promise for increasing KI efficiency. Combined use of these reagents with genome editing components might improve KI efficiency in *i*-GONAD-based genome editing. Notably, simultaneous introduction of three kinds of gRNA, called “triple-target CRISPR”^35^, may increase the efficiency of indels in our *i*-GONAD-based system. According to Sunagawa *et al*.^35^, the efficiency of indel generation increased up to 98% when triple-target CRISPR was employed, compared with 36~65% efficiency achieved by introduction of a single gRNA.

In conclusion, we succeeded in producing genome-edited (KO and KI) rats using *i*-GONAD. In addition to the rat strain and locus used in Kobayashi *et al*.^32^, we showed that *i-*GONAD can also be applicable to several other rat strains such as a total of five rat strains including three pure stains (BN, SD and LEW), and two types of F1 hybrid rats between BN and SD and other locus such as the *Pax6* locus in addition to the *Tyr* locus. We tried to modify *Pax6* locus beside *Tyr* locus, whereas Kobayashi *et al*.^32^ did *Tyr* locus alone. We also assessed possible improvement to KI efficiency by employing SCR7 although few improvement was produced. Lastly, we provide evidence for germ-line transmission of genome-edited traits produced through the *i*-GONAD system (see Fig. 3). We confidently predict that this technology will make it possible to create genome-edited animals in other mammals, such as hamster and pig.

## Methods

### Animals

Slc:SD (SD), BN/SsNSlc (BN), and LEW/SsNSlc (LEW) rats were purchased from Japan SLC, Inc. (Shizuoka, Japan). Adult rats between the ages of 8–10 weeks (female) and 10–12 weeks (male) were used. All rats were maintained under temperature-controlled conditions (24 ± 2 °C) with a 12 L/12D light cycle (lights on at 7:00 AM). A solid diet and water were provided *ad libitum*. The animal facility was maintained under specific pathogen-free (SPF) conditions. This study was approved by the *Hamamatsu University School of Medicine Animal Care and Use Committee* (permission no. 2017-064 and 2017-100). We performed all animal experiments in accordance with the Law for the Humane Treatment and Management of Animals (Japanese Law No. 105), Standard Relating to the Care and Management of Laboratory Animals and Relief of Pain (Notice No. 6 of the Prime Minister’s Office), and the Guidelines for Proper Conduct of Animal Experiments (Science Council of Japan).

### Estrus cycle monitoring

Vaginal smears were collected each morning (8:00–9:00 am) using a cotton tipped swab wetted with distilled water to determine estrus cyclicity of each rat. The swab was inserted into a rat vagina and gently turned and rolled against the vaginal wall. The cells of the vaginal canal were transferred to a dry glass slide by rolling the swab across the slide. The slide was air dried and then fixed with methanol, and stained with Giemsa stain solution for 30 min at room temperature. The slides were rinsed with water, air dried, and viewed immediately at 200× magnification under bright field illumination. The stage of the estrus cycle was determined based on the presence or absence of leukocytes, cornified epithelial cells, and nucleated epithelial cells according to Felicio *et al*.^36^. Cell classification was based on the stages of the rat estrus cycle (proestrus, estrus, metestrus and diestrus)^37^. Female rats in proestrus phase were mated at 17:00–18:00 pm to sexually experienced male rats (over 10 weeks of age). Mating strategies were as follows: BN females were mated to SD males; SD females were mated to SD or BN males; and LEW females were mated to LEW males. The following morning, females with sperm in their vaginal smear were judged as pregnant females and used for *i*-GONAD experiments.

### Preparation of *i*-GONAD solutions

For preliminary tests in rats, we used a 20 µL mixture containing 0.5 mg/mL EGFP mRNA (Miltenyi Biotec, Tokyo, Japan), 1 mg/mL Rhodamine (3 kDa, Thermo Fisher, MA, USA), and 0.02% Fast Green (Nacalai tesque, Kyoto, Japan) dissolved in Opti-MEM® medium (Thermo Fisher Scientific).

For CRISPR/Cas9-mediated induction of indels, Alt-R® CRISPR-Cas9 crRNAs were designed to recognize target sites [exon 2 of rat *Tyr* (Figs 1E and 2B) or exons 2 and 3 of rat *Pax6* (Fig. 4A)] that match a 20 bp DNA sequence (Supplementary Table S8) just upstream of the PAM. crRNAs were synthesized by Integrated DNA Technologies, Inc. (IDT) (IA, USA). Alt-R® CRISPR-Cas9 tracrRNA was also purchased from IDT. Each reagent was dissolved in Opti-MEM® medium at a final concentration of 100 μM, and stored at −80 °C until use. crRNAs and tracrRNA were annealed by mixing equimolar amounts of each (30 μM) and incubating at room temperature for about 10 min to allow formation of crRNA:tracrRNA duplexes. The annealed crRNA:tracrRNA duplexes (6 μL) were mixed with Cas9 protein (10 μg/μL; purchased from IDT) to form RNP complexes in Opti-MEM® medium in a 0.5 mL PCR tube. Thus, the final concentrations of components were 30 µM crRNA:tracrRNA duplexes, 1 μg/μL Cas9 protein and 0.02% Fast Green dissolved in Opti-MEM® medium.

For CRISPR/Cas9-mediated KI using an ssODN, the ssODN with 64 bp (left arm) and 65 bp (right arm) homologous arms at both ends was custom DNA synthesized (Macrogen, Seoul, South Korea) (Supplementary Table S8). The lyophilized ssODN was re-suspended in Opti-MEM® to the concentration of 10 µg/µL. Annealed crRNA:tracrRNA duplexes (3 μL each) were mixed with Cas9 protein (1 μL) and ssODN (2 μL) in Opti-MEM® to a total of 10 μL in a 0.5 mL PCR tube, so that the final concentrations of components were 30 µM crRNA:tracrRNA duplexes, 1 μg/μL Cas9 protein, 2 µg/µL ssODN and 0.02% Fast Green.

For CRISPR/Cas9-mediated KI with SCR7 (Xcess Biosciences, Inc., San Diego, CA, USA), a potent and selective inhibitor of NHEJ (Srivastava *et al*.)^24^, SCR7 was dissolved in dimethyl sulfoxide (Sigma-Aldrich, MI, USA) at a final concentration of 50 mM, and stored at −20 °C until use. SCR7 (1 or 10 µM final concentration) was added to 10 μL Opti-MEM® containing RNP, ssODN and Fast Green.

### *i*-GONAD procedure

In mice, *i*-GONAD is usually performed at 0.7 dpc (corresponding to the late 1-cell stage; at 16:00 pm of the day when copulatory plugs are confirmed), because eggs at this stage are thought to be free from cumulus cells (Fig. 1A), which may hamper efficient uptake of intraoviductally instilled CRISPR/Cas9-related components^16^. Rat embryos at 0.7 dpc (females with sperm in their vaginal smear on the morning after mating with a male is defined as 0 dpc) correspond to late 1-cell mouse embryos^38^. Thus, we performed *i*-GONAD at 0.7 dpc (16:00 pm).

Surgical procedures were performed on adult females anesthetized with a mix of three anesthetic agents (medetomidine, midazolam, and butorphanol)^39^ under a dissecting microscope, as described previously^14–16^ with slight modifications. The ovary/oviduct/uterus were exposed after making an incision (~2 cm in length) in the dorsal skin and a subsequent incision (~1 cm in length) in the muscle layer. The exposed ovary, oviduct and part of the uterus were placed on a small piece of paper towel (about 2–3 cm^2^) and adipose tissue was anchored with an Aorta-Klemme to prevent return of the exposed tissues. Approximately 1.5 μL of solution was injected into the oviduct lumen upstream of the ampulla using a micropipette (prepared using an electric puller; PN-3; NARISHIGE, Tokyo, Japan) and an attached mouthpiece (Fig. 1B-a–d). Immediately after the injection, the oviduct regions were covered with a piece of wet paper soaked in Dulbecco’s modified phosphate buffered saline (DPBS), and grasped in tweezer-type electrodes (#CUY652-3; NEPA GENE, Chiba, Japan) (Fig. 1B-e). Electroporation was performed using a square-wave pulse generator NEPA21 (NEPA GENE Co. Ltd.). The electroporation parameters were as follows: Poring pulse (Pp); 50 V, 5 msec pulse, 50 msec pulse interval, 3 pulses, 10% decay (±pulse orientation) and Transfer pulse (Tp); 10 V, 50 msec pulse, 50 msec pulse interval, 3 pulses, 40% decay (±pulse orientation). After electroporation, oviducts were returned to their original position, the incisions made in the internal dorsal muscle were sutured, and the dorsal skin was closed using a surgical stapler. The animals were then given an intradermal detoxicant ANTISEDAN® (Nippon Zenyaku Kogyo, Fukushima, Japan), monitored for anesthesia recovery, and housed for further analysis.

For injection with a mixture of EGFP mRNA, Rhodamine and Fast Green, two groups were prepared: one group (denoted as Exp [experimental group]) received intraoviductal instillation of a solution and subsequent *in vivo* electroporation, and the other group (denoted as Cont [control group]) received intraoviductal instillation of a solution only, without electroporation.

### Treatment of the *i*-GONAD-derived offspring

Pregnant female rats at 1.5 or 14.5 to 16.5 dpc were subjected to euthanasia by overdose of intraperitoneal sodium pentobarbital. Embryos (2-cell) were isolated from oviducts by a flushing method described by Hogan *et al*.^40^ and inspected for fluorescence under a fluorescence microscope. 14.5 to 16.5 dpc fetuses were dissected out in DPBS. Pigmentation in eyes and morphological abnormality were assessed under a dissecting microscope and photographed. Tail biopsies were then taken for genomic DNA isolation from mid-gestational fetuses. In some cases, pregnant females were allowed to deliver their pups, and coat pigmentation was inspected and photographed 4 weeks after birth. Tail biopsies were also taken for genomic DNA isolation from pups.

### Analysis of CRISPR/Cas9-induced mutations and KI

The on-target and potential off-target regions were analysed by PCR and sequencing. The potential off-target regions were searched for using the CHOPCHOP website (http://chopchop.cbu.uib.no/index.php).

Genomic DNA was isolated from tail biopsies of mid-gestational fetuses or newborn rats by incubation in 100 μL 50 mM NaOH at 95 °C for 10 min. Then, 10 μL 1 M Tris-HCl (pH 8.0) was added to the aliquot and mixed. This crude DNA extract was used as template for PCR.

PCR amplification of an on-target locus (*Tyr* or *Pax6*) and off-target locus (Supplementary Table S7) were performed in 20 µL containing 10 µL 2x PCR buffer for KOD FX, 0.4 mM dNTPs, 1 µL crude lysate, 0.25 µM primer pairs (Supplementary Table S8), and 0.1 U KOD FX (TOYOBO, Osaka, Japan) under cycling conditions of, denaturation (94 °C for 3 min), (amplification) 33 cycles of 95 °C for 20 sec, 57 °C for 30 sec and 68 °C for 1 min, and extension (68 °C for 5 min). Amplification products (5 μL) were separated by 2% agarose gel electrophoresis.

PCR products were directly sequenced using the dideoxy chain termination method with a BigDye Terminator v3.1 Cycle Sequencing kit (Thermo Fisher Scientific), and then analysed on an automated ABI PRISM 3100 DNA sequencer (Thermo Fisher Scientific). In some cases, PCR products were sub-cloned into the pTA cloning system for sequencing. PCR products with blunt ends from 3′-5′ exonuclease activity of KOD polymerase, were given overhanging dA at the 3′ ends using 10x A-attachment mix (TOYOBO), and then sub-cloned into the pMD20-T vector (TAKARA, Shiga, Japan) prior to sequencing.

### Observation of fluorescence

Recovered embryos were observed using a fluorescence stereomicroscope fitted with a filter for detecting EGFP (EVOS® FLoid® Cell Imaging Station; Thermo Fisher Scientific Inc.) and Rhodamine-derived fluorescence.

## Electronic supplementary material

Supplemental information
