# Supplementary material for: i-GONAD (improved genome-editing via oviductal nucleic acids delivery), a convenient in vivo tool to produce genome-edited rats
Source: Sci Rep. 2018 Aug 13;8:12059. doi: 10.1038/s41598-018-30137-x (PMC6089882; doi:10.1038/s41598-018-30137-x)
Supplement: Supplementary file 1 — Supplemental information [file 41598_2018_30137_MOESM1_ESM.pdf]

i-GONAD (improved genome-editing via oviductal nucleic acids delivery), a convenient *in vivo* tool to produce genome-edited rats

Shuji Takabayashi, Takuya Aoshima, Katsuya Kabashima, Kazushi Aoto, Masato Ohtsuka, Masahiro Sato

Supplementary Table S1. Exp-1: *i*-GONAD-mediated induction of indels in fetuses of BSF1 (F1 hybrid between BN female and SD male).

Supplementary Table S2. Exp-2: *i*-GONAD-mediated induction of indels in fetuses of SBF1 (F1 hybrid between SD female and BN male).

Supplementary Table S3. Exp-3: *i*-GONAD-mediated KI into the *Tyr* locus in fetuses/newborn of LEW rats.

Supplementary Table S4. Exp-4: *i*-GONAD-mediated KI into the *Tyr* locus in fetuses/newborn of SD rats.

Supplementary Table S5. Exp-5: KI into the *Tyr* locus in SD fetuses/newborn offspring obtained after *i*-GONAD in the presence of 1 μM SCR7.

Supplementary Table S6. Exp-5: KI into the *Tyr* locus in SD fetuses/newborn offspring obtained after *i*-GONAD in the presence of 10 μM SCR7.

Supplementary Table S7. Mutations at putative off-target loci containing up to 3 bp mismatches for target gene are analyzed by sequencing.

Supplementary Table S8. Nucleotide sequences of oligonucleotides (oligo) used in this study.

Supplemental Fig. S1. Sequencing of the junction between endogenous *Tyr* and the ssODN using KI rats (LEW-#16 and SD-#55).

Supplemental Fig. S2. Genotyping of the unedited tails [isolated from SD, BN and offspring (C1-C3) obtained after mating between SD and BN) or fetal samples (Pax6-#4 and #5 shown in Fig. 4B-D)]. The PCR condition is the same shown in Fig. 4D. Note that there is no additional band throughout these samples, except for the presence of a single band at 1,489 bp.

Supplemental Fig. S3. Sequencing of the PCR products (derived from abnormal fetuses carrying indels in the target *Pax6* locus; shown in **Fig. 4D**) cloned into a TA cloning vector. Based on these sequence data, the deduced amino acid sequences are aligned and shown in **Fig. 4F**.

Supplemental Fig. S4. Sequencing results of off-target candidate loci in fetal/newborn offspring after *i*-GONAD. The 20-bp target sequences and PAM (underlined) are shown.

Supplementary Table S1. Exp-1: *i*-GONAD-mediated induction of indels in fetuses of BSF1 (F1 hybrid between BN female and SD male).

Name of pregnant females	Name of fetuses	Eye color	Genotype	Note for the target *Tyr* sequence
#1	BSF1-#1	Black	Normal (G/A)	-
#1	BSF1-#2	Black	Normal (G/A)	-
#1	BSF1-#3	White	Homozygous bi-allelic (A/A)	Transition of G to A in allele-G
#1	BSF1-#4	White	Heterozygous mono-allelic (del)	Deletion of (TTACGTA) in allele-G
#1	BSF1-#5	White	Heterozygous mono-allelic (del)	Deletion of (TTA) in allele-G
#1	BSF1-#6	White	Heterozygous mono-allelic (indels)	Deletion of 146-bp sequence (CATTGTATGTAATTTCATTCATATGTAAGTCCCTTGTCTCAGAGATAACTCATTTTGCATAAATTGGTTTTCACAGATCATTTGCAGCAGATCAGAAGAGTATAATAGCCATCAGGTTTTATGTGATGGAACACCTGAGGGACCAC) and insertion the sequence (AAACATGAAAA) is newly added in allele-G
#1	BSF1-#7	White	Homozygous bi-allelic (in)	Insertion of (TTT) in both alleles, as shown by CTA(TTT)TTA
#2	BSF1-#8	Black	Normal (G/A)	-
#2	BSF1-#9	White	Heterozygous mono-allelic (in)	Insertion of T in allele-G, as shown by TATT(T)ACG
#2	BSF1-#10	White	Heterozygous mono-allelic (del)	Deletion of (TATTAC) in allele-G (see Fig. 1G)
#3	BSF1-#11	Black	Normal (G/A)	-
#3	BSF1-#12	Black	Normal (G/A)	-
#3	BSF1-#13	White	Heterozygous mono-allelic (del)	Deletion of (CCACTA) in allele-G.

Abbreviations: in, insertion mutation; del, deletion mutation; indels, insertion and deletion mutations.

Supplementary Table S2. Exp-2: *i*-GONAD-mediated induction of indels in fetuses of SBF1 (F1 hybrid between SD female and BN male).

Name of pregnant females	Name of fetuses	Eye color	Genotype	Note for the target *Tyr* sequence
#1	SBF1-#14	Black	Normal (G/A)	-
#1	SBF1-#15	Black	Normal (G/A)	- (see Fig. 1G)
#1	SBF1-#16	White	Heterozygous mono-allelic (del)	Deletion of 89-bp sequence (AAATTGGTTTTCACAGATCATTTGCAGCAGATCAGAAGAGTATAATAGCCATCAGGTTTTATGTGATGGAACACCTGAGGGACCACTAT) in allele-G
#2	SBF1-#17	Black	Normal (G/A)	-
#3	SBF1-#18	Black	Normal (G/A)	-
#3	SBF1-#19	Black	Normal (G/A)	-
#3	SBF1-#20	Black	Normal (G/A)	-
#3	SBF1-#21	Black	Normal (G/A)	-
#3	SBF1-#22	White	Heterozygous mono-allelic (del)	Deletion of 51-bp sequence (TGAGGGACCACTATTACGTAATCCTGGAAACCATGACAAAGCCAAAACCCC) in allele-G
#3	SBF1-#23	White	Heterozygous mono-allelic (del)	Deletion of 214-bp sequence (TCCTCCCTTCACTTTCATCTAACTGCTAGGAAGCTGAATGATAATAATGATATGTAATTTCATTGTATGTAATTTCATTCATATGTAAGTCCCTTGTCTCAGAGATAACTCATTTTGCATAAATTGGTTTTCACAGATCATTTGCAGCAGATCAGAAGAGTATAATAGCCATCAGGTTTTATGTGATGGAACACCTGAGGGACCACTATTACGT) in allele-G
#3	SBF1-#24	White	Heterozygous mono-allelic (in)	Insertion of (AA) in allele-G, as shown by CTA(AA)TTACG
#3	SBF1-#25	White	Compound heterozygous bi-allelic (indels)	Deletion of (TTACGTA) in allele-G, and insertion of (CA) in the allele-A, as shown by CTA(CA)TTACA
#3	SBF1-#26	White	Heterozygous mono-allelic (in)	Insertion of (T) in allele-G, as shown by TATT (T)ACG
#3	SBF1-#27	White	Mosaic (in)	Allele-A is normal, but there are deletion of 15-bp (TTACGTAATCCTGGA) sequence in allele-G, and deletion of 3-bp (TTA) sequence in the other allele-G
#3	SBF1-#28	White	Mosaic (indels)	Allele-A is normal, but there are insertion of (T) in allele-G, as shown by TATT(T)ACG, and deletion of 7-bp (TTACGTA) sequence in the other allele-G
#3	SBF1-#29	White	Mosaic (indels)	One allele is allele-A, but there are insertion of (TA) in the other allele-A, as shown by TATTA(TA)CAT, and deletion of 22-bp sequence (ACTATTACGTAATCCTGGAAAC) in the other allele-G

Abbreviations: in, insertion mutation; del, deletion mutation; indels, insertion and deletion mutations.

Supplementary Table S3. Exp-3: *i*-GONAD-mediated KI into the *Tyr* locus in fetuses/newborn of LEW rats.

Name of pregnant females	Name of fetuses	Eye color	Genotype	Note for the target *Tyr* sequence
#1	LEW-#1	White	Normal (A/A)	-
#1	LEW-#2	White	Normal (A/A)	-
#2	LEW-#3	White	Compound heterozygous bi-allelic (indels)	Insertion of GA in one allele, as shown by ACCACTA(GA)TTACAT, and deletion of 103-bp sequence (ATAATCCTGGAAACCATGACAAAGCCAAAACCCCCAGGCT CCCATCTTCAGCAGACGTGGAATTTTGTCTGAGTTTGACCC AGTATGAATCTGGATCAATGGA) in the other allele
#2	LEW-#4	White	Heterozygous mono-allelic (in)	Insertion of (TT) in one allele, as shown by ACCACTATT(TT)ACATA
#2	LEW-#5	White	Normal (A/A)	-
#2	LEW-#6	White	Heterozygous mono-allelic (in)	Insertion of (A) in one allele, as shown by ACCACTA(A)TTACAT (see Fig. 2D)
#3	LEW-#7	White	Normal (A/A)	-
#3	LEW-#8	White	Normal (A/A)	-
#3	LEW-#9	White	Normal (A/A)	-
#3	LEW-#10	White	Normal (A/A)	-
#3	LEW-#11	White	Normal (A/A)	-
#4	LEW-#12	White	Compound heterozygous bi-allelic (indels)	Deletion of (TTA) in one allele, and deletion of (TTAC) and insertion of (A) in the other allele, as shown by ACCACTA~~TTAC~~(A)ATAAT
#4	LEW-#13	White	Normal (A/A)	-
#4	LEW-#14	White	Heterozygous mono-allelic (replacement)	Replacement of CCCAACTAAACCTTGAGC by TAATAGCCATCAGGTTT in one allele
#4	LEW-#15	White	Heterozygous mono-allelic (in)	Insertion of (TT) in one allele, as shown by ACCACTATT(TT)ACA
#5	LEW-#16	Black	KI (G)/ mosaic (in)	Successful KI of ssODN in one allele (allele-G) (see Fig. 2D and Supplemental Fig. S1), insertion of A in the other allele (allele-A), as shown by ACCACTA(A)TTACA, and insertion of CA in one more another allele (allele-A'), as shown by ACCACTA(CA)TTACA
#5	LEW-#17	White	Normal (A/A)	-
#5	LEW-#18	White	Normal (A/A)	-
#5	LEW-#19	White	Normal (A/A)	-
#5	LEW-#20	White	Mosaic (indels)	One allele is allele-A, but there are insertion of (TT) in one allele, as shown by ACCACTATT(TT)ACA, and deletion of 23-bp sequence (CTATTACATAATCCTGGAAACCA) in the other allele
#5	LEW-#21	White	Compound heterozygous bi-allelic (indels)	Insertion of (T) in one allele, as shown by ACCACTATT(T)ACA and deletion of 21-bp sequence (CCACTATTACATAATCCTGGA) in the other allele
#5	LEW-#22	White	Normal (A/A)	-

Abbreviations: in, insertion mutation; del, deletion mutation; indels, insertion and deletion mutations.

Supplementary Table S4. Exp-4: *i*-GONAD-mediated KI into the *Tyr* locus in fetuses/newborn of SD rats.

Name of pregnant females	Name of fetuses	Eye color	Genotype	Note for the target *Tyr* sequence
#1	SD-#1	White	Normal (A/A)	-
#1	SD-#2	White	Normal (A/A)	-
#1	SD-#3	White	Normal (A/A)	-
#1	SD-#4	White	Normal (A/A)	-
#1	SD-#5	White	Heterozygous mono-allelic (in)	Insertion of (A) in one allele, as shown by ACCACTA(A)TTACA
#1	SD-#6	White	Normal (A/A)	-
#1	SD-#7	White	Mosaic (in)	One allele has albino-specific allele A (allele-A). Insertion of A in another allele, as shown by ACCACTA(A)TTACA (allele-A'), and insertion of (AT) in the other allele, as shown by ACCACTA(AT)TTACA (allele-A'')
#1	SD-#8	White	Heterozygous mono-allelic (KI and in)	Successful KI of ssODN and insertion of (ATTACTAA) in allele-G, as shown by CCACTA(ATTACTAA)TTACG
#1	SD-#9	White	Heterozygous mono-allelic (in)	Insertion of (AATA) in one allele, as shown by ACCACTA(AATA)TTACA
#1	SD-#10	White	Normal (A/A)	-
#1	SD-#11	White	Compound heterozygous bi-allelic (in)	Insertion of (A) in one allele, as shown by ACCACTA(A)TTACA, and insertion of (TA) in the other allele, as shown by ACCACTA(TA)TTACA
#2	SD-#12	White	Normal (A/A)	-
#2	SD-#13	White	Normal (A/A)	-
#2	SD-#14	White	Normal (A/A)	-
#2	SD-#15	White	Mosaic (indels)	One allele has albino-specific allele A (allele-A). Insertion of (TT) in another allele (allele-A'), as shown by ACCACTATT(TT)ACA, deletion of 41-bp sequence (ACTATTACATAATCCTGGAAACCATGACAAAGCCAAA ACCC) in the other allele (allele-A''), and deletion of 31-bp sequence (GACCACTATTACATAATCCTGGAAACCATGA) in one more another allele (allele-A''')
#2	SD-#16	White	Mosaic (in)	One allele has albino-specific allele A (allele-A). Insertion of (AA) in another allele (allele-A'), as shown by ACCACTA(AA)TTACA, and insertion of (T) in the other allele (alleles A''), as shown by ACCACTATT(T)ACA
#2	SD-#17	White	Normal (A/A)	-
#2	SD-#18	White	Homozygous bi-allelic (KI and in)	Successful KI of ssODN and three insertions in both alleles, as shown by GTGAT(T)GGAACACCT(G)AGGACCACTATTAC<u>G</u>TAATC C(C)TGGAAA
#2	SD-#19	White	Heterozygous mono-allelic (del)	Deletion of 6-bp sequence (CCACTAT) in one allele
#2	SD-#20	White	Mosaic (indels)	Insertion of A in one allele (allele-A), as shown by ACCACTA(A)TTACA, insertion of TT in another allele (allele-A'), as shown by ACCACTATT(TT)ACA, and deletion of 7-bp sequence (ACATAAT) in one more another allele (allele-A'')
#2	SD-#21	White	Normal (A/A)	-
#2	SD-#22	White	Normal (A/A)	-
#2	SD-#23	White	Heterozygous mono-allelic (in)	Insertion of (A) in one allele, as shown by ACCACTA(A)TTACA
#3	SD-#24	White	Normal (A/A)	-
#3	SD-#25	White	Normal (A/A)	-
#3	SD-#26	White	Compound heterozygous bi-allelic (del)	Deletion of 6-bp sequence (TATTAC) in one allele, and deletion of 7-bp sequence (TATTACA) in the other allele
#4	SD-#54	Black	Heterozygous KI (G/A)	Successful KI of ssODN in one allele (see Fig. 2F)

#4	SD-#55	Black	KI (G)/ heterozygous mono-allelic (indels)	Successful KI of ssODN in one allele (allele-G; see Supplemental Fig. S1) and deletion of (T)/insertion of (A) in the other allele, as shown by ~~T~~ATGTGATGGAACACCTGAGGGACC(A) (allele-A)
#4	SD-#56	White	Heterozygous mono-allelic (in)	Insertion of (T) in one allele, as shown by ACCACTATT(T)ACA.
#4	SD-#57	White	Normal (A/A)	-
#4	SD-#58	White	Mosaic (indels)	One allele has albino-specific allele A (allele-A). There are insertion of (AA) in the other allele (allele-A'), as shown by ACCACTA(AA)TTACA, and deletion of 13-bp sequence (ATTACATAATCCT) in one more another allele (allele-A'')
#4	SD-#59	White	Normal (A/A)	-
#4	SD-#60	White	Normal (A/A)	-
#4	SD-#61	White	Normal (A/A)	-
#4	SD-#62	White	Normal (A/A)	-
#4	SD-#63	White	Normal (A/A)	-
#4	SD-#64	White	Normal (A/A)	-
#4	SD-#65	White	Normal (A/A)	-
#4	SD-#66	White	Normal (A/A)	-
#4	SD-#67	White	Heterozygous mono-allelic (in)	Insertion of (AA) in one allele, as shown by ACCACTA(AA)TTACA

Abbreviations: in, insertion mutation; del, deletion mutation; indels, insertion and deletion mutations.

Supplementary Table S5. Exp-5: KI into the *Tyr* locus in SD fetuses/newborn offspring obtained after *i*-GONAD in the presence of 1 μM SCR7.

Name of pregnant females	Name of fetuses	Eye color	Genotype	Note for the target *Tyr* sequence
#1	SD-#27	Black	KI (G)/ mosaic (indels)	Successful KI of ssODN in one allele (allele-G). There are deletion of 7-bp sequence (ACATAAT) in another allele (allele-A'), and insertion of (A) in one more another allele (allele-A''), as shown by ACCACTA(A)TTACA
#1	SD-#28	White	Normal (A/A)	-
#1	SD-#29	White	Heterozygous mono-allelic (del)	Deletion of 2-bp sequence (TA) in one allele.
#2	SD-#30	White	Normal (A/A)	-
#2	SD-#31	White	Normal (A/A)	-
#2	SD-#32	White	Normal (A/A)	-
#2	SD-#33	White	Normal (A/A)	-
#2	SD-#34	White	Normal (A/A)	-
#2	SD-#35	White	Normal (A/A)	-
#3	SD-#36	White	Normal (A/A)	-
#3	SD-#37	White	Normal (A/A)	-
#3	SD-#38	White	Normal (A/A)	-
#4	SD-#39	White	Compound heterozygous bi-allelic (indels)	Deletion of (T) in one allele, as shown by ACCACTA~~T~~TACA and deletion of (TTAC)/insertion of (A) in the other allele, as shown by ACCACTA~~TTAC~~(A)ATAA
#5	SD-#40	White	Heterozygous mono-allelic (in)	Insertion of (TT) in one allele, as shown by ACCACTATT(TT)ACA
#5	SD-#41	White	Heterozygous mono-allelic (in)	Insertion of (TT) in one allele, as shown by ACCACTATT(TT)ACA
#5	SD-#42	White	Normal (A/A)	-
#5	SD-#43	White	Normal (A/A)	-
#5	SD-#44	White	Mosaic (indels)	One allele has albino-specific allele A (allele-A). There are insertion of (A) in another allele (allele-A'), as shown by ACCACTA(A)TTACA, and deletion of 23-bp sequence (TATTACATAATCCTGGAAACCAT) in one more another allele (allele-A'')
#5	SD-#45	White	Mosaic (indels)	One allele has albino-specific allele A (allele-A). There are insertion of (A) in another allele (allele-A'), as shown by ACCACTA(A)TTACA, and deletion of (CTATTACA) in one more another allele (allele-A'')
#6	SD-#46	Black	KI (G)/ mosaic (in)	Successful KI of ssODN in one allele (allele-G). There are insertion of (TT) in another allele (allele-A'), as shown by ACCACTATT(TT)ACA, and insertion of (A) in one more another allele (allele-A''), as shown by ACCACTA(A)TTACA
#6	SD-#47	White	Homozygous bi-allelic (del)	Deletion of 96-bp sequence (CATAAATTGGTTTTCACAGATCATTTGCAGCAGATCA GAAGAGTATAATAGCCATCAGGTTTTATGTGATGGAAC ACCTGAGGGACCACTATTACA) in both alleles
#6	SD-#48	White	Normal (A/A)	-
#6	SD-#49	White	Normal (A/A)	-
#6	SD-#50	White	Normal (A/A)	-
#6	SD-#51	White	Normal (A/A)	-
#6	SD-#52	White	Mosaic (indels)	One allele has albino-specific allele A (allele-A). There are insertion of (T) in another allele (allele-A'), as shown by ACCACTATT(T)ACA, and deletion of 7-bp sequence (TTACATA) in one more another allele (allele-A'')

#6	SD-#53	White	Mosaic (indels)	One allele has albino-specific allele A (allele-A). There are insertion of (TT) in another allele (allele-A'), as shown by ACCACTATT(TT)ACA, and deletion of 115-bp sequence (ATTACATAATCCTGGAAACCATGACAAAGCCAAAACCCCCAGGCTCCCATCTTCAGCAGACGTGGAATTTTGTCTGAGTTTGACCCAGTATGAATCTGGATCAATGGATAGAACT) in one more another allele (allele-A'')

Abbreviations: in, insertion mutation; del, deletion mutation; indels, insertion and deletion mutations.

Supplementary Table S6. Exp-5: KI into the *Tyr* locus in SD fetuses/newborn offspring obtained after *i*-GONAD in the presence of 10 µM SCR7.

Name of pregnant females	Name of fetuses	Eye color	Genotype	Note for the target *Tyr* sequence
#1	SD-#68	White	Compound heterozygous bi-allelic (del)	Deletion of (TAT) in one allele, as shown by ACCAC~~TAT~~TACA and deletion of (TATTAC) in the other allele, as shown by ACCAC~~TATTAC~~ATAA
#1	SD-#69	White	Normal (A/A)	-
#1	SD-#70	White	Normal (A/A)	-
#1	SD-#71	White	Mosaic (indels)	One allele has albino-specific allele A (allele-A). There are insertion of (AT) in another allele (allele-A'), as shown by ACCACTAT(AT)TACA, and deletion of (T) in one more another allele (allele-A''), as shown by ACCACTAT~~T~~ACA.
#1	SD-#72	White	Normal (A/A)	-
#1	SD-#73	White	Normal (A/A)	-
#1	SD-#74	White	Heterozygous mono-allelic (in)	Insertion of (CCTGAGGGACCAC) in one allele, as shown by ACCACTA(CCTGAGGGACCAC)TTACA
#1	SD-#75	Black	KI (G)/ mosaic (in)	Successful KI of ssODN in one allele (allele-G). One allele has albino-specific allele A (allele-A). Deletion of 5-bp sequence (TACAT) in one more another allele (allele-A''), as shown by ACCACTAT~~TACAT~~AAT (see Fig. 3B).
#2	SD-#76	Black	KI (G)/ mosaic (in)	Successful KI of ssODN in one allele (allele-G). There are insertion of (C) in another allele (allele-A'), as shown by ACCACTA(C)TTACA, and deletion of 5-bp sequence (CTATTA) in one more another allele (allele-A''), as shown by ACCA~~CTATTA~~CATAAT.
#2	SD-#77	White	Compound heterozygous bi-allelic (indels)	Deletion of (TTACATAATCCTGG) in one allele, as shown by ACCACTA~~TTACATAATCCTGG~~AAACCA and deletion of (ACTA)/insertion of (TGGAAA) in the other allele, as shown by ACC~~ACTA~~(TGGAAA)TTACATAAT.
#2	SD-#78	White	Homozygous bi-allelic (del)	Deletion of (TACAT) in both alleles, as shown by ACCACTAT~~TACAT~~AATCCTGG.
#2	SD-#79	White	Normal (A/A)	-
#2	SD-#80	White	Normal (A/A)	-
#2	SD-#81	White	Mosaic (indels)	Deletion of (TTACA) in one allele (allele-A), as shown by ACCACTA~~TTACA~~TAAT and insertion of (A) in the other allele (allele-A'), as shown by ACCACTA(A)TTACATAAT, and deletion of (CTATTACA) in one more another allele (allele-A"), as shown by ACCA~~CTATTACA~~TAAT.
#2	SD-#82	White	Mosaic (indels)	Insertion of A in one allele (allele-A), as shown by ACCACTA(A)TTACA, insertion of A in another allele (allele-A'), as shown by ACCACTA(A)TTACA, and deletion of (CTA) in one more another allele (allele-A"), as shown by ACCA~~CTA~~TTACA.
#2	SD-#83	White	Normal (A/A)	-
#2	SD-#84	White	Normal (A/A)	-
#2	SD-#85	White	Normal (A/A)	-
#3	SD-#86	White	Normal (A/A)	-
#3	SD-#87	White	Normal (A/A)	-
#3	SD-#88	White	Heterozygous mono-allelic (in)	Insertion of (AT) in one allele, as shown by ACCACTA(AT)TTACATAAT

Abbreviations: in, insertion mutation; del, deletion mutation; indels, insertion and deletion mutations.

Supplementary Table S7. Mutations at putative off-target loci containing up to 3 bp mismatches for target gene are analyzed by sequencing.

Name	Sequence (5'-3')	Locus	Gene name	Mutation	Primer-F	Primer-R
Tyr-wild-crRNA	TTTCCAGGATTACGTAATAGTGG	chr1 : +151097594	Tyr	12/12	GCTCAAGGTTTAGTTGGGTACT	CTGGCTAGGTTTACTATCTCCTTG
W-offtarget 1	TTTCCAGGAAAAGGTAATAGTGG	chr14 : +37687330	Fryl	0/12	ATCAGCACTAGACTGGTCAA	GAGAGTCTGTCACAAGCTTC

Name	Sequence (5'-3')	Locus	Gene name	Mutation	Primer-F	Primer-R
Tyr-mutant-crRNA	TTTCCAGGATTATGTAATAGTGG	chr1 : +151097594	Tyr	12/12	GCTCAAGGTTTAGTTGGGTACT	CTGGCTAGGTTTACTATCTCCTTG
M-offtarget 1	TTTCCAGAACTATGTAATACTGG	chr11 : +30871276	-	0/12	GAGTGAGCAATGGCGGATAC	CACAGTTGCTCACACGCTTT
M-offtarget 2	TTTCCAGGATTTTGTGCTAGTGG	chr11 : +36928561	-	0/12	TACTGGGGAGGTACAAGGAG	AGCATCTCCAAGGATGGGT
M-offtarget 3	TTTCCAGGAAAAGGTAATAGTGG	chr14 : +37687330	Fryl	0/12	ATCAGCACTAGACTGGTCAA	GAGAGTCTGTCACAAGCTTC
M-offtarget 4	TTTCCACGAATATGTAAAAGTGG	chr8 : +90874461	-	0/12	CAGCTCAAGCTTCTGGGATT	CGTGAGGATCTGGTTCTGTTG

Name	Sequence (5'-3')	Locus	Gene name	Mutation	Primer-F	Primer-R
Pax6-crRNA1	GGAGGATCACCTGCAGAATTCGG	chr3 : -95715312	Pax6	4/4	CCTCCTTTCCTCAGGTCACA	GGAGACCCATGCAAATCAGG
P1-offtarget 1	GAAGGATCAGCTGCAGAATGCGG	chr5 : +134499947	Cyp4a1	0/4	TCCTCTAACCCTACCCAATCC	CAGGACACTCTGAACTTCCTC

Name	Sequence (5'-3')	Locus	Gene name	Mutation	Primer-F	Primer-R
Pax6-crRNA2	GGATGTGTGAGTAAAATTCTGGG	chr3 : +95716248	Pax6	4/4	CCTCCTTTCCTCAGGTCACA	GGAGACCCATGCAAATCAGG
P2-offtarget 1	GGATGTTAGAGTCAAATTCTGGG	chr1 : -32441545	-	0/4	ATGTCCACAACAGCAGACAG	GTGGCTAAGACATACCACAACC
P2-offtarget 2	GGATGTGTGGGTACATTTCTGGG	chr14 : +47851159	-	0/4	GTCCTTTAGCCACTTGGACTT	GTGCAATAATGCTGGAGGATG
P2-offtarget 3	GCATGTGTGAGAAATATTCTGGG	chr15 : +27165193	-	0/4	GTGGCAACTTTTGGAAGTCTG	AGAATGTTTCCCCTCTCGATG
P2-offtarget 4	TGATATGTGACTAAAATTCTGGG	chr9 : +94093203	Dis3l2	0/4	CTCATGAGTGTGAGGTCAGTC	CTAAGTCTGCTCCCACATC

Mismatches compared to on-target sequence are shown in red; PAM sequences are labeled in blue.

Supplementary Table S8. Nucleotide sequences of oligonucleotides (oligo) used in this study.

Oligo-type	Sequence (5'-3')	Sequence (5'-3')
crRNA	Tyr-wild-crRNA	TTT CCA GGA TTA CGT AAT AG (TGG)
	Tyr-mutant-crRNA	TTT CCA GGA TTA TGT AAT AG (TGG)
	Pax6-crRNA1	GGA GGA TCA CCT GCA GAA TT(CGG)
	Pax6-crRNA2	GGA TGT GTG AGT AAA ATT CT(GGG)
ssODN	Tyr wild ssODN	AGA TCA GAA GAG TAT AAT AGC CAT CAG GTT TTA TGT GAT GGA ACA CCT GAG GGA CCA CTA TTA CGT AAT CCT GGA AAC CAT GAC AAA GCC AAA ACC CCC AGG CTC CCA TCT TCA GCA GAC GTG GAA TTT T
Primer	On-target primer	
	Rat Tyr-F	GCT CAA GGT TTA GTT GGG TAC T
	Rat Tyr-R	CTG GCT AGG TTT ACT ATC TCC TTG
	Rat Pax6-F	CCT CCT TTC CTC AGG TCA CA
	Rat Pax6-R	GGA GAC CCA TGC AAA TCA GG
	Off-target primer	
	W-offtarget 1 -F	ATC AGC ACT AGA CTG GTC AA
	W-offtarget 1 -R	GAG AGT CTG TCA CAA GCT TC
	M-offtarget 1 -F	GAG TGA GCA ATG GCG GAT AC
	M-offtarget 1 -R	CAC AGT TGC TCA CAC GCT TT
	M-offtarget 2 -F	TAC TGG GGA GGT ACA AGG AG
	M-offtarget 2 -R	AGC ATC TCC AAG GAT GGG T
	M-offtarget 3 -F	ATC AGC ACT AGA CTG GTC AA
	M-offtarget 3 -R	GAG AGT CTG TCA CAA GCT TC
	M-offtarget 4 -F	CAG CTC AAG CTT CTG GGA TT
	M-offtarget 4 -R	CGT GAG GAT CTG GTT CTG TTG
	P1-offtarget 1 -F	TCC TCT AAC CCT ACC CAA TCC
	P1-offtarget 1 -R	CAG GAC ACT CTG AAC TTC CTC
	P2-offtarget 1 -F	ATG TCC ACA ACA GCA GAC AG
	P2-offtarget 1 -R	GTG GCT AAG ACA TAC CAC AAC C

Primer	P2-offtarget 2 -F	GTC CTT TAG CCA CTT GGA CTT
	P2-offtarget 2 -R	GTG CAA TAA TGC TGG AGG ATG
	P2-offtarget 3 -F	GTG GCA ACT TTT GGA AGT CTG
	P2-offtarget 3 -R	AGA ATG TTT CCC CTC TCG ATG
	P2-offtarget 4 -F	CTC ATG AGT GTG AGG TCA GTC
	P2-offtarget 4 -R	CTA AGT CTG CTC CCA CAT C

Parentheses indicate PAM sequence in crRNA.

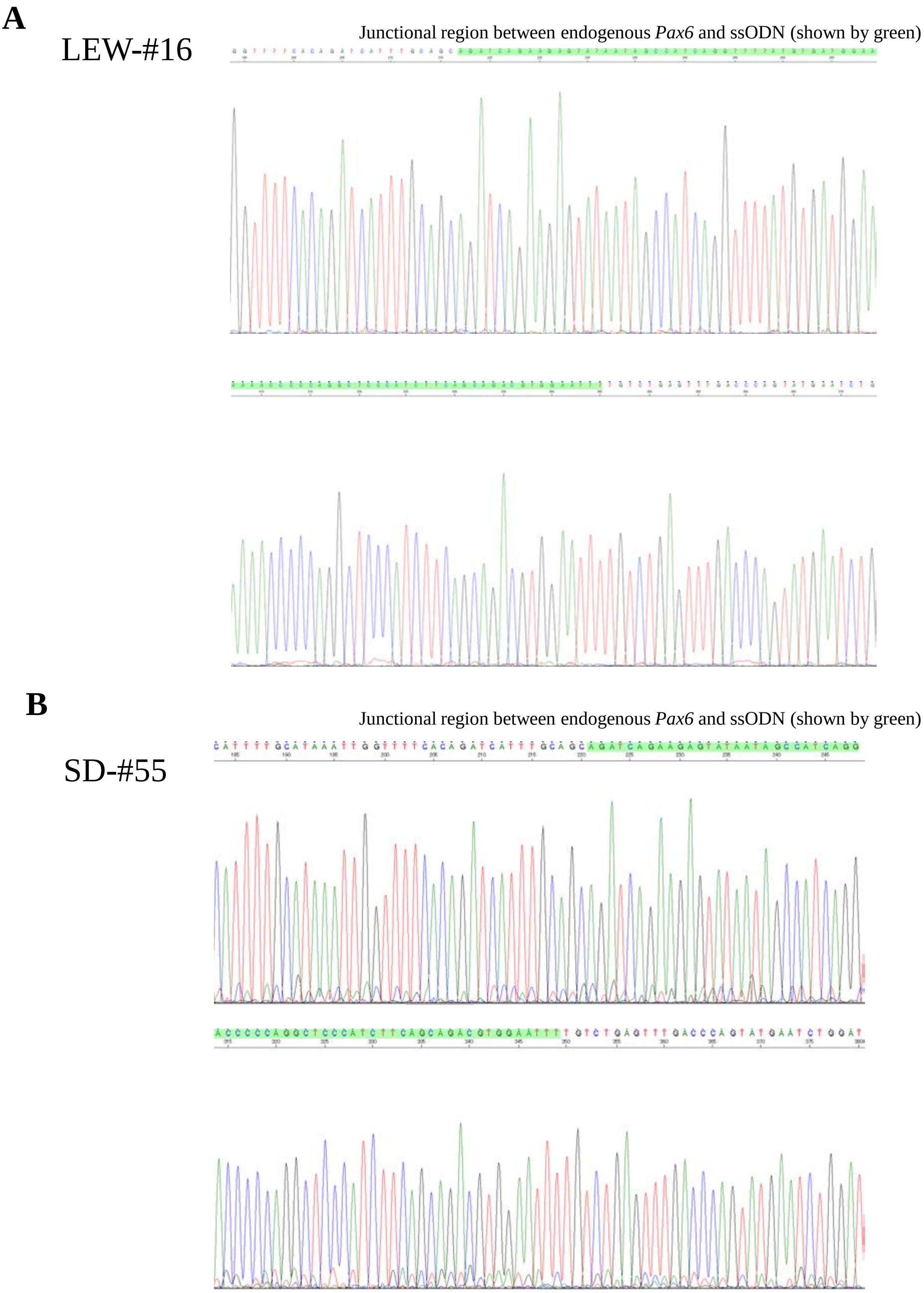

Supplemental Fig. S1. Sequencing of the junction between endogenous *Tyr* and the ssODN using KI rats (LEW-#16 and SD-#55).

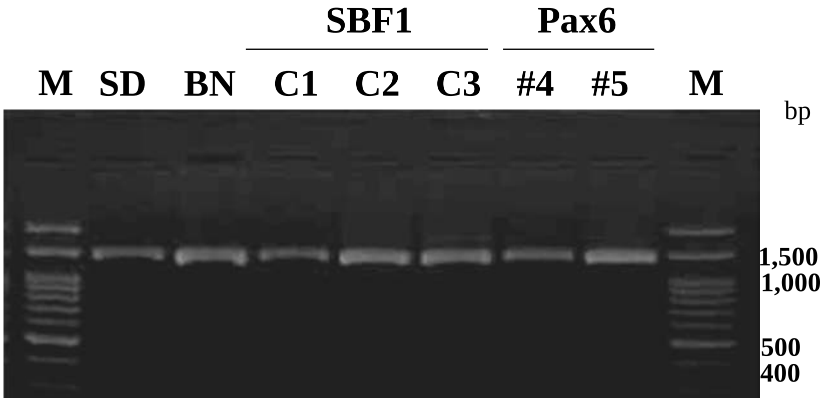

Supplemental Fig. S2. Genotyping of the unedited tails [isolated from SD, BN and offspring (C1-C3) obtained after mating between SD and BN) or fetal samples (Pax6-#4 and #5 shown in Fig. 4B-D)]. The PCR condition is the same shown in Fig. 4D. Note that there is no additional band throughout these samples, except for the presence of a single band at 1,489 bp.

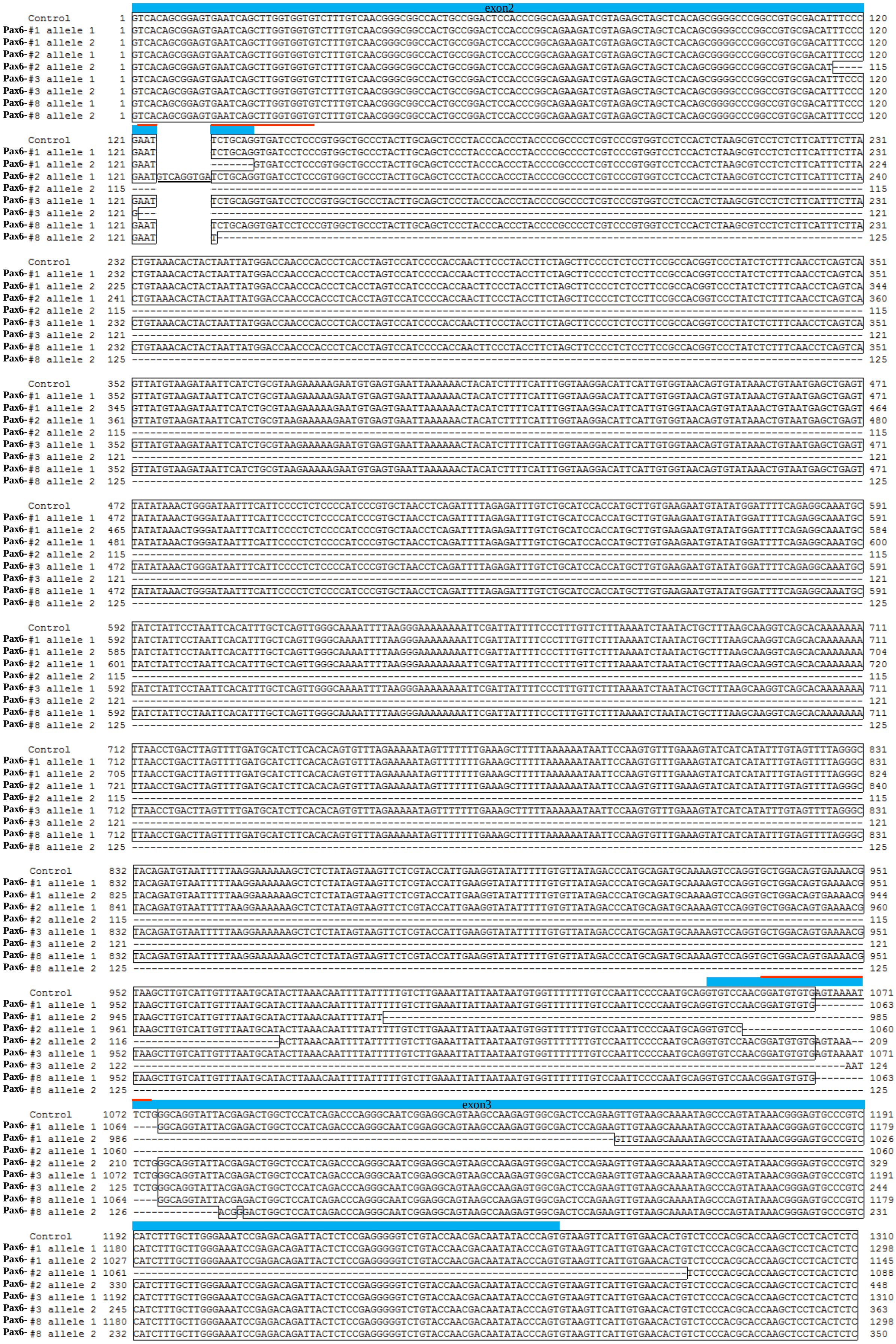

Supplemental Fig. S3. Sequencing of the PCR products (derived from abnormal fetuses carrying indels in the target *Pax6* locus; shown in **Fig. 4D**) cloned into a TA cloning vector. Based on these sequence data, the deduced amino acid sequences are aligned and shown in **Fig. 4F**.

W-off target 1

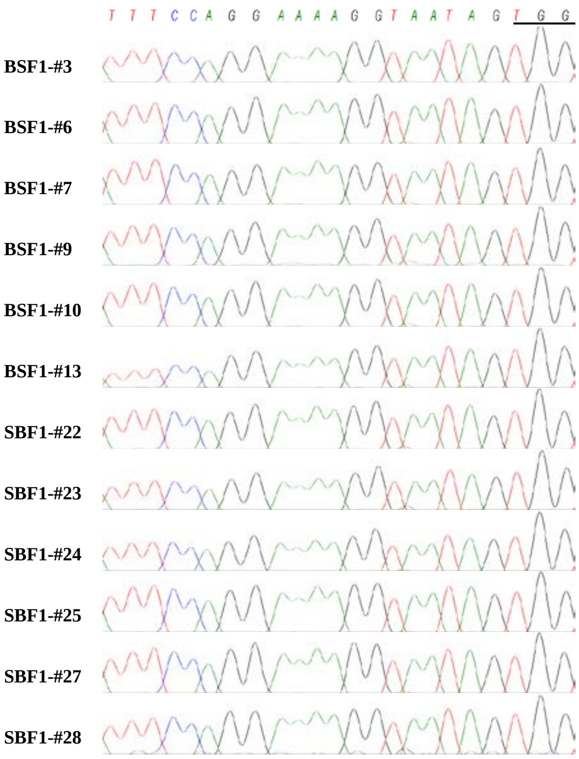

M-off target 1

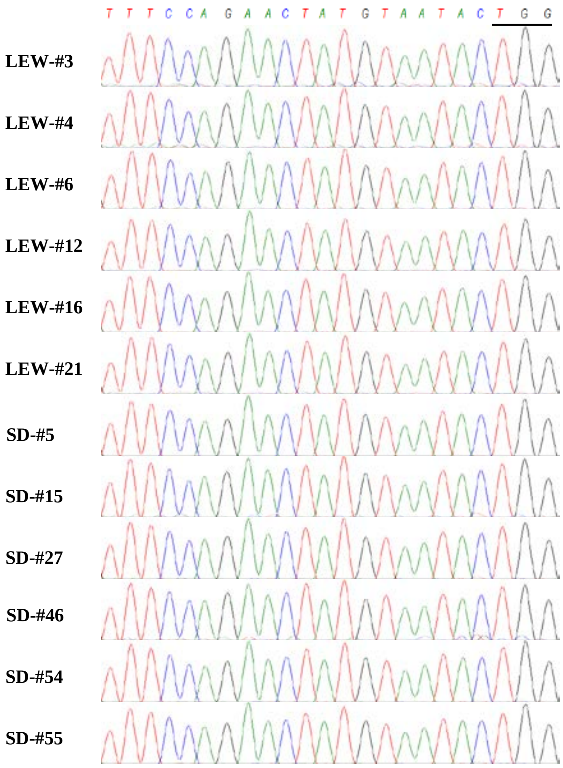

M-off target 2

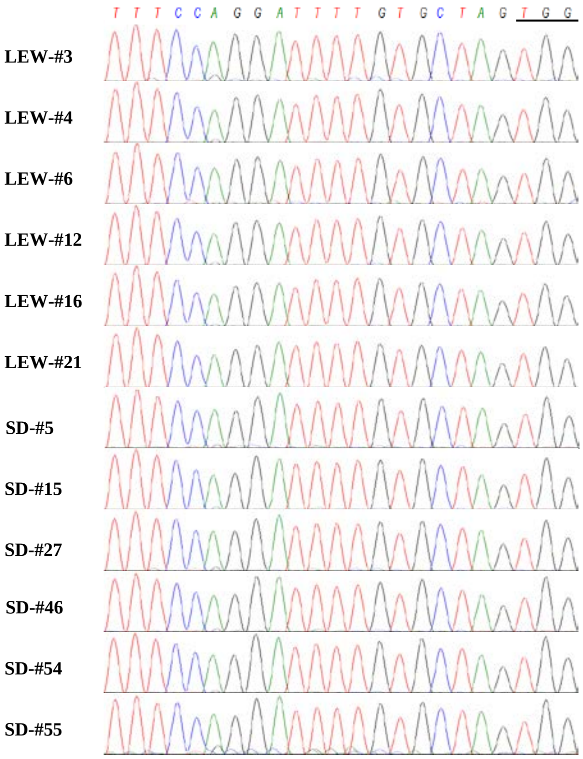

M-off target 3

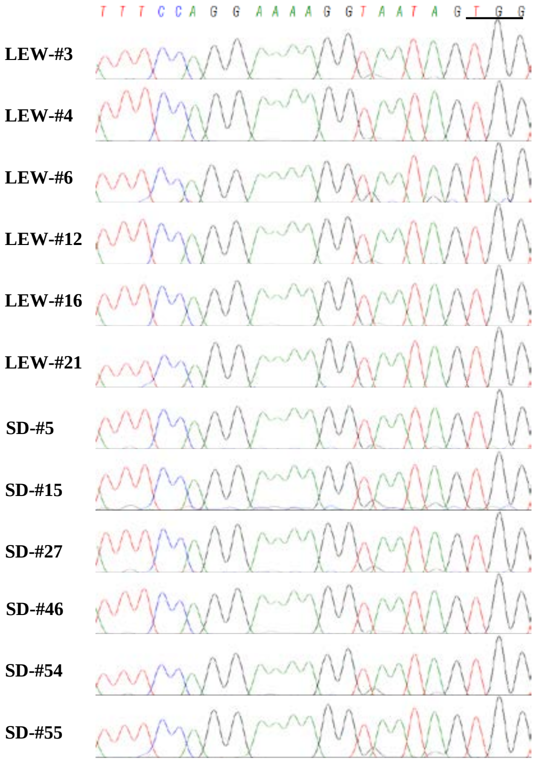

Supplemental Fig. S4

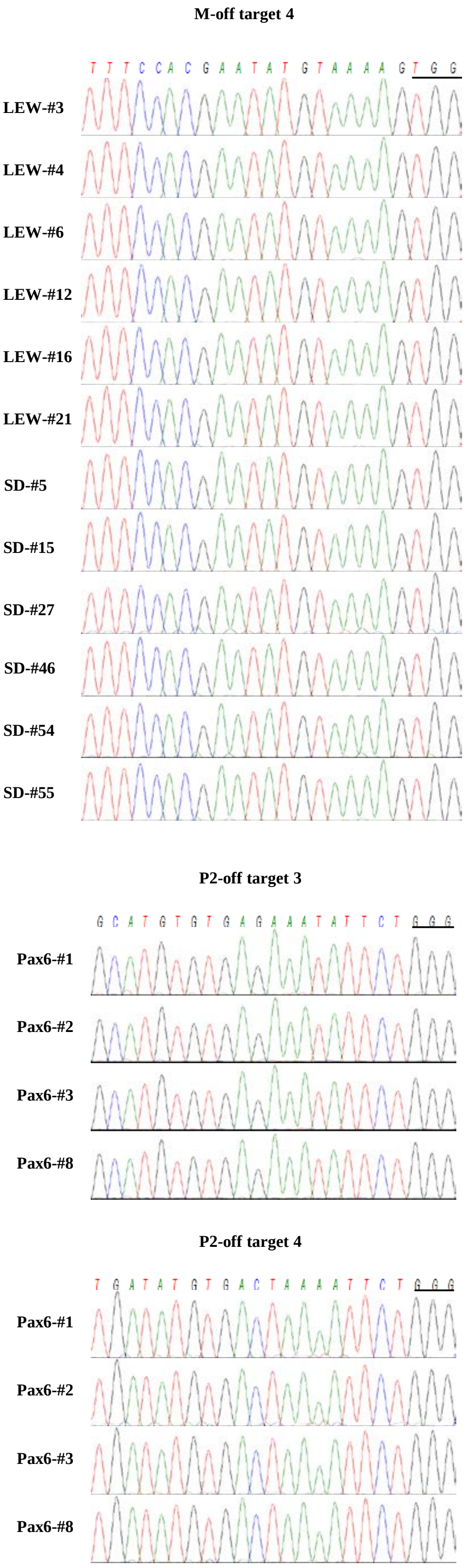

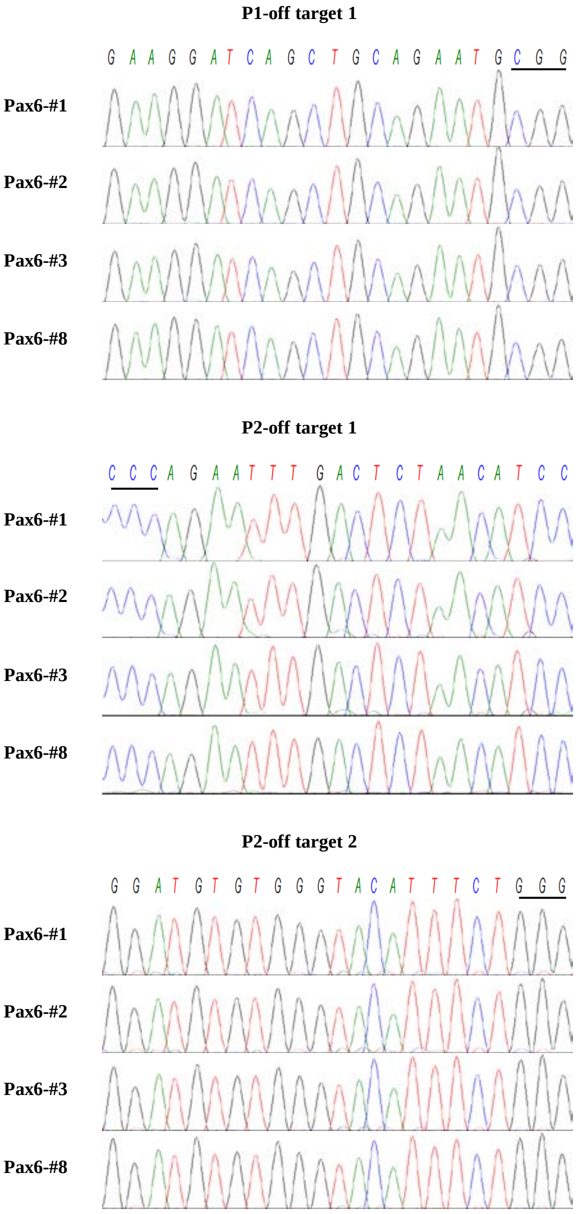

Supplemental Fig. S4. Sequencing results of off-target candidate loci in fetal/newborn offspring after *i*-GONAD. The 20-bp target sequences and PAM (underlined) are shown.
